# Extracellular Vesicles in Allergy: From Cellular Communication to Clinical Implications

**DOI:** 10.1007/s12016-026-09161-7

**Published:** 2026-04-29

**Authors:** Angelika Sysak, Sabina Górska

**Affiliations:** 1 Laboratory of Microbiome Immunobiology, Hirszfeld Institute of Immunology and Experimental Therapy, Polish Academy of Sciences, Wrocław, Poland; 2 Department of Pharmacology and Toxicology, Faculty of Veterinary Medicine, Wrocław University of Environmental and Life Sciences, Wrocław, Poland

**Keywords:** Extracellular vesicle, Allergy, Asthma, Atopic dermatitis, Food allergy, Exosome

## Abstract

Extracellular vesicles (EVs) are lipid bilayer-enclosed particles released by both eukaryotic and prokaryotic cells and represent an evolutionarily conserved system of intercellular communication. By transporting bioactive cargo, including proteins, lipids, microRNAs, EVs enable the transfer of molecular signals between cells, thereby regulating immune homeostasis and inflammatory responses. In allergic diseases, EVs have emerged as key mediators linking epithelial barriers, immune cells, and the microbiome. EVs derived from epithelial, immune, and microbiota-associated cells may contribute to the initiation, amplification, and persistence of allergic inflammation by modulating barrier integrity, immune cell polarization, and cytokine signaling pathways. Disease-specific alterations in EV cargo reflect underlying pathogenic mechanisms, positioning EVs as promising non-invasive biomarkers for disease diagnosis, stratification, and monitoring. In parallel, accumulating experimental evidence highlights the therapeutic potential of EVs as cell-free immunomodulatory agents capable of suppressing allergic inflammation and promoting immune tolerance. This review synthesizes current knowledge on extracellular vesicles across three major allergic diseases: asthma, atopic dermatitis, and food allergy, integrating mechanistic insights with diagnostic and therapeutic advances. By incorporating highly recent literature and covering a broad spectrum of EV sources and engineered vesicle-based strategies, the review provides a comprehensive overview of how EV-mediated cellular communication translates into clinically relevant applications in allergy.

## Introduction

One of the earliest descriptions of vesicle-like structures derived from platelets was reported in 1967 by Wolf, who identified lipid-rich particles in human plasma and serum referred to as “platelet dust”. Little was known about its physicochemical properties at the time, apart from its lipid-rich structure and coagulant properties similar to those of platelet factor 3 [1]. Intensive development of analytical methods has allowed for significant progress in research aimed at determining the properties and functions of extracellular vesicles (EVs). The Minimal Information for Studies of Extracellular Vesicles (MISEV) [2] guidelines were established by the International Society for Extracellular Vesicles to standardize research on EVs. MISEV provides recommendations on EVs isolation, characterization, and reporting, aiming to ensure reproducibility, reliability, and comparability of results across studies. By following these guidelines, researchers can produce high-quality, well-documented data, facilitating the advancement of the EVs field. For example, the size distribution and concentration of EVs may be measured using Nanoparticle Tracking Analysis, which tracks the Brownian motion of particles in a liquid suspension, or Dynamic Light Scattering, which analyses the scattering of light [3, 4]. To examine the detailed structure and morphology of EVs, the most commonly employed techniques include Transmission Electron Microscopy, Atomic Force Microscopy, and Confocal Microscopy [3–5]. Flow Cytometry enables quantification and phenotypic characterization of EVs, including subtype determination [6]. In particular, imaging cytometry has emerged as a highly useful tool for EVs characterization, allowing simultaneous sizing, enumeration, and phenotyping [7, 8]. To characterise the molecular content of EVs, Western Blotting and Enzyme-Linked Immunosorbent Assay (ELISA) are commonly used for specific protein detection, while Mass Spectrometry (MS) and Raman Spectroscopy provide information about protein and lipid content [9–11]. Quantitative reverse transcription PCR (qRT-PCR) serves as a standard method to assess specific RNA cargo, including mRNA and microRNA (miRNA) [12]. Recently developed Single-Particle Interferometric Reflectance Imaging Sensor facilitates EVs detection and profiling across unpurified and purified sources, yielding information on size, number, phenotype, and biomarker distribution [13].

Because EVs are produced by both eukaryotic and prokaryotic cells, this phenomenon is considered as evolutionary link, indicating their importance for the functioning and survival of both types of cells. Various criteria are used for the classification of EVs, for example, their functions, size, cargo included, or mechanism of biosynthesis. EVs are surrounded by a spherical membrane made from a lipid bilayer, and apart from the cytosol, they contain various biologically active molecules of the parental cell origin, such as proteins (cytokines, growth and transcription factors, receptors), genetic material including miRNAs, long non-coding RNAs, and mRNA [14]. EVs origin and biogenesis are strictly connected with their size, composition, and biological functions. They are usually classified into three main classes: exosomes (with a diameter between 30 nm and 150 nm), microvesicles (100–1000 nm), and apoptotic bodies (50–5000 nm) [15]. Importantly, EV composition and function depend on their cellular origin and the methods used for their isolation and analysis, which may significantly influence study outcomes. This heterogeneity should be considered when interpreting experimental data, particularly in translational contexts.

EVs are now recignized as key mediators of intercellular communication, actively modulating immune responses, and thus involved in numerous physiological and pathological processes [16]. In the context of allergic diseases, EVs derived from immune and epithelial cells may contribute to the initiation and amplification of inflammation by transferring bioactive molecules that regulate immune cell activation and signaling pathways [17]. Their cargo reflects disease-specific processes, making them promising candidates as biomarkers for diagnosis, disease monitoring, and therapeutic targeting [18]. Despite these insights, the precise roles of EVs in allergic pathophysiology remain incompletely understood, underscoring the need for a comprehensive synthesis of current knowledge. Unlike prior reviews that focus on a single disease or a narrow mechanistic scope, this manuscript provides an integrated perspective on asthma, atopic dermatitis, and food allergy within a unified framework. It bridges mechanistic insights with current diagnostic and therapeutic strategies. Particular emphasis is placed on microbiome-derived extracellular vesicles, an emerging and often underexplored area in allergy research, as well as on the translational potential of EVs as non-invasive biomarkers and cell-free immunomodulatory agents.

## Role and Mechanism of Action of Extracellular Vesicles in the Development of Allergic Diseases

### Extracellular Vesicles in the Development of Allergic Asthma

Asthma is an inflammatory disease affecting approximately 300 million people worldwide and is characterized by airway hyperresponsiveness, mucus overproduction, and airway remodeling. Its pathogenesis is driven by complex interactions between epithelial and immune cells, including Th2 lymphocytes, dendritic cells (DCs), mast cells, and eosinophils, which together orchestrate type 2 inflammation [19]. Airway remodeling observed in the course of asthma is a complex process that involves structural, cellular, and molecular alterations. One of them is thickening of the subepithelial extracellular matrix (ECM) with increased collagen deposition (collagens I, II, and VI) accompanied by goblet cell hyperplasia and smooth muscle hypertrophy [20]. Furthermore, complex interactions between epithelial cells, fibroblasts, and mast cells within proinflammatory hubs occur in spatially-organized niches that produce alarmins and chemokines [21]. One of the most critical remodeling factor is epithelial cell-derived lumican acting by promoting fibroblast proliferation and collagen production while the balance between matrix metalloproteinases and tissue inhibitors determines whether remodeling proceeds through ECM deposition in acute models or ECM degradation in chronic inflammation models. Airway remodeling is also characterized by apoptosis of infiltrating immune cells in acute models localized to alveolar regions, and by necroptosis in structural cells during chronic remodeling, which releases pro-inflammatory mediators and accelerates innate immune responses [22]. Bronchoalveolar lavage fluid (BALF) remains a gold standard for assessing airway inflammation, enabling analysis of airway and alveolar components and provides valuable insights into disease mechanisms through proteomic and genomic analyses [23].

Altered EVs profiles observed in BALF from asthmatic patients compared to healthy controls suggest that they actively contribute to airway inflammation and asthma pathogenesis rather then merely reflecting it [24]. One of the key mechanisms by which EVs modulate asthma pathogenesis is the transfer of microRNAs, which regulate gene expression in recipient cells primarily through mRNA degradation or translational inhibition [25]. They are involved in numerous biological processes such as lung development, immune responses, and the pathogenesis of pulmonary diseases such as lung cancer, asthma, chronic obstructive pulmonary disease (COPD), and pulmonary fibrosis [26]. The functions of extracellular vesicle miRNAs in allergic diseases are summarized in Table 1.

**Table 1:** Role of extracellular vesicle miRNAs in allergic diseases

Extracellular vesicle miRNAs	Origin	Experimental design	Outcomes	Ref.
↑ miR-125b-5p ↑ miRNA-126 ↑ miR-122-5p	Serum of asthmatic patients	Human asthma	↑ airways obstruction	[27–29]
↑ miR-21-5p ↑ miR-126-3p ↑ miR146a-5p ↑ miR-215-5p	Serum of asthmatic patients	Human asthma	↑ Th2 differentiation	[30]
↓miR-21-5p ↓ miR-126-3p ↓ miR146a-5p	Serum of older asthmatic patients with obesity		↑ neutrophil and basophil counts ↑ TNF	
↑ miR-134-5p ↑ miR-207 ↑ miR-465-5p ↑ miR-30b-5p ↑ miR-19a-3p ↑ miR-130a-3p	Rat serum	ZnO- induced neutrophilic asthma rat model	↑ pulmonary inflammation	[31]
↑ miR-346	Bronchoalveolar lavage fluid (BAL)	House dust mite (HDM)-induced murine asthma model	↑ eosinophilic infiltration ↑ IL-13	[32]
↓ miR-92b ↓ miR-34a ↓ miR-210	Human bronchial epithelial cells (HBEC)	HBEC stimulated in vitro with IL-13	↑ dendritic cell maturation ↑ Th2 response	[33]
	Nasal lavage	Childhood asthma	↑ airways obstruction	
↑ miR-320 ↑ miR-181 ↑ miR-550 ↑ let-7 ↑ miR-154	HBEC- basolateral side	Human asthma	↑ T and B cell receptors signalling pathways	[34]
↑ let-7i-5p	HBEC	HBEC stimulated in vitro with PM2.5	↑ MAPK pathway activation ↑ bronchoconstriction in a murine asthma model	[35]
miR-181a miR-17 mir-155	Human breast milk	Pathway analysis	↑ number of Foxp3^+^ CD4^+^ CD25^+^ regulatory T cells in PBMC ↑ B cell differentiation	[36]
let-7-5p miR-148a-3p miR-30-5p miR-200a-3p miR-141-3p miR-22-3p miR-181-5p miR-146b-5p miR-378a-3p miR-29-3p miR-200b/c-3p miR-429-3p	Human breast milk	Pathway analysis	Immune development and tolerance establishment by: TGF-beta signalling T cell receptor signalling Toll-like receptor signalling JAK-STAT signalling Th1 and Th2 cell differentiation PI3K-Akt, MAPK, TNF, and FoxO signaling	[37]
miRNA-150	CD8^+^ suppressor T (Ts) lymphocytes of mice tolerized to casein	Delayed-type food hypersensitivity to casein murine model	↓ inflammatory response	[38]
miR-146a-5p	Human induced pluripotent stem cell-derived mesenchymal stem cells (iPSC-MSCs)	IL-33 induced eosinophilic airway inflammation murine model	↓ group 2 innate lymphoid cells (ILC2) levels ↓ inflammatory infiltration ↓ mucus secretion ↓ Th2 cytokines (IL-5, IL-13) ↓ airway hyperresponsiveness	[39]
miR-301a-3p	Rat adipose-derived MSC (ADSC)	Airway smooth muscle cells stimulated *in vitro* with platelet-derived growth factor (PDGF-BB)	↓ proliferation and migration ↓ TNF-α, IL-1β, IL-6 through STAT3 pathway	[40]
		OVA-induced murine asthma model	↓ airway fibrosis ↓ IL-1β, IL-6, TNF-α, MCP-1 in lung tissue	
miR-146a-5p	Human umbilical cord-derived MSC (hUCMSCs)	OVA-induced murine asthma model	↓ total inflammatory cells ↓ eosinophils ↓ IL-4 and IL-13 in BALF ↓ airway remodeling	[41]
		Human lung fibroblasts (HLF-1) stimulated in vitro with TGF-β1	↓expression of profibrotic markers p-smad2/3, α-smooth muscle actin (α-SMA), collagen-1 ↓ fibrogenic pathways responsible for airway remodeling	
miR-511-3p	HEK-293T-derived EVs decorated with RNA nanotechnology-based PRNA-3WJ nanoparticles	Cockroach allergen-induced murine asthma model	↓ inflammatory infiltration ↓ goblet cell hyperplasia ↓ BAL inflammatory cells ↓ eosinophils ↓ Th2 cytokines (IL-4, IL-5, IL-13) ↑ macrophage M2 polarization	[42]
hsa-miR-4517	Human airway epithelial cells (AEC, cell line A549) stimulated with *Micrococcus luteus*- derived EVs	Monocytes derived from the blood of asthmatic patients stimulated in vitro with LPS	↓ IL-1β ↓ NLRP3 expression ↓ group 3 innate lymphoid cells (ILC3) activation ↓ neutrophil recruitment	[43]
miR-100-5p	Pearl oyster *Pinctada martensii* mucus	AD murine model	↓ collagen synthesis ↓expression of FOXO3 and NLRP3 signaling pathway ↓ inflammation	[44]

**Fig. 1:**
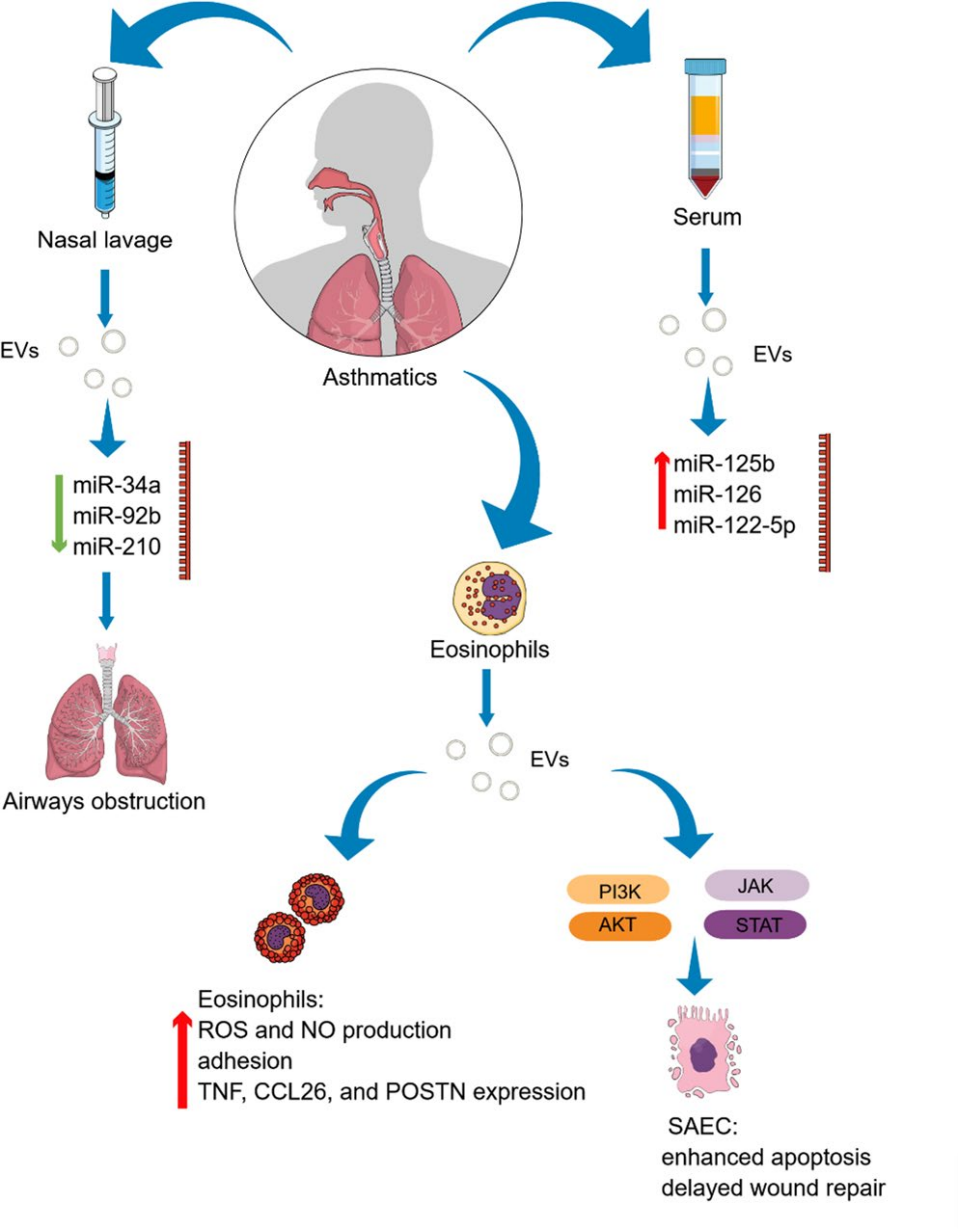
EVs derived from asthmatic patients contribute to the progression of the disease. Reduced levels of miR-34a, miR-92b, and miR-210 in extracellular vesicles isolated from nasal lavages of children with asthma, compared with healthy controls, are associated with airway obstruction [33]. EVs isolated from serum also differ in miRNAs content in asthmatics and healthy donors. The levels of miRNA-125b-5p, miRNA-126 and miR-122-5p molecules are elevated and correlate with more severe airflow obstruction in asthmatic patients [27–29]. Eosinophils modulate their own activity via the secretion of extracellular vesicles (EVs). EVs isolated from blood eosinophils of patients with asthma increase the production of reactive oxygen species (ROS) and nitric oxide (NO), enhance adhesion to fibronectin, and promote eosinophil survival. In addition, these EVs induce apoptosis in human small airway epithelial cells (SAEC) and delay wound repair by modulating the phosphoinositide 3-kinase/protein kinase B(PI3K/AKT) and Janus kinase–signal transducer and activator of transcription (JAK-STAT) signaling pathways [53, 54]

The presence of miRNAs within airway-derived EVs was first demonstrated by Sinha et al., who identified exosome-enclosed miRNAs in exhaled breath condensate (EBC) from asthmatic patients, highlighting their diagnostic potential [45, 46]. Since then, numerous miRNAs have been linked to asthma pathogenesis. For instance, miRNA-125b-5p has been associated with inflammatory responses in asthma, with lower expression in bronchial biopsy correlating with more severe airflow obstruction in asthmatic patients [47]. This miRNA was also identified in serum-derived exosomes from patients with asthma, where its expression was elevated and positively correlated with disease severity [27] (Table 1; Fig. 1). Similar observations were reported by Atashbasteh et al. who demonstrated increased expression of miR-125b in the plasma exosomal fraction of patients with severe asthma which correlated with inflammatory indices measured in serum, including IgE and hs-CRP levels [48]. The occurrence of opposite correlations between allergic disease severity and miRNA levels in EVs isolated from serum and tissues may be explained by several biological mechanisms. First, cells are able to selectively package specific miRNAs into EVs and release them into circulation, resulting in increased EV miRNA levels in serum and decreased EV miRNA levels in extracellular environment. Second, the export of regulatory miRNAs via EVs may serve as a mechanism to reduce their intracellular concentration or to facilitate intercellular communication by modulating signaling pathways in recipient cells. Finally, allergic inflammation may alter EV biogenesis and cargo selection, thereby influencing the enrichment of specific miRNAs in EVs and leading to disease-specific expression patterns [49, 50]. Metabolomic and cytokine profiling studies further support the concept that local airway alterations are reflected systemically. Changes observed in BALF and lung tissue, including metabolic dysregulation and cytokine shifts, are often mirrored in serum, indicating that EV-associated signals can propagate beyond the lung environment [51]. Interestingly, Hogea et al. showed correlation between the levels of cytokines in serum and BALF in patients with lung cancer and chronic lung disease [52]. Cytokine levels were significantly elevated in patients with malignancies, and analysis of BALF showed higher concentrations than in serum. Notably, cancer-associated cytokines appeared earlier and at greater levels in lavage fluid than in peripheral blood. This clearly shows that changes observed in BALF or lung tissue are frequently reflected in the serum, indicating that local pulmonary alterations, including shifts in EVs profile, can manifest systemically. Therefore, EVs obtained noninvasively from nasal lavage or serum may serve as a minimally invasive “liquid biopsy,” providing valuable information on disease status and progression through biomarker analysis.

**Fig. 2:**
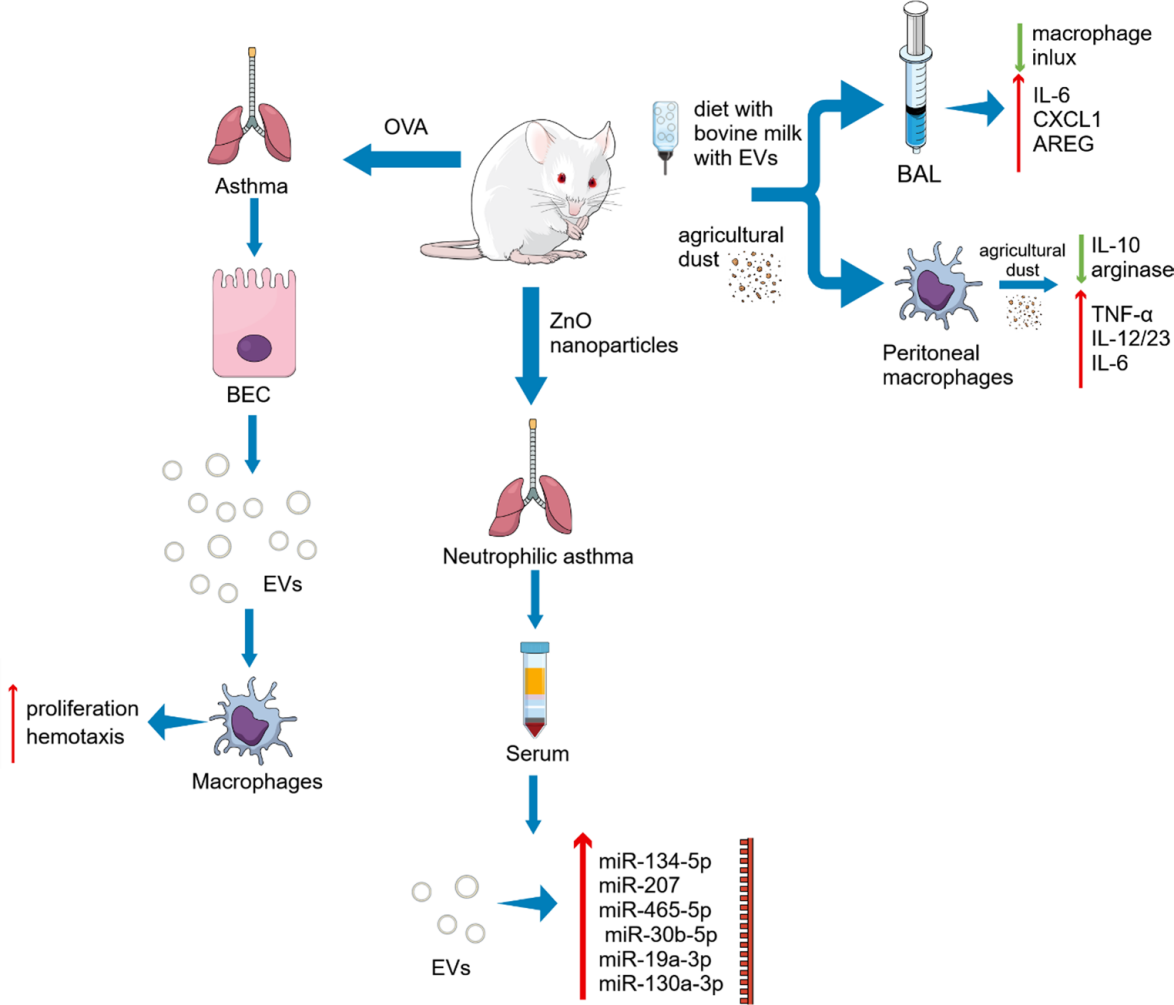
Animal models used to elucidate the contribution of extracellular vesicles (EVs) to airway allergic inflammation. Pulmonary neutrophilic inflammation induced by zinc oxide nanoparticles in rats resulted in increased levels of miR-134-5p, miR-207, miR-465-5p, miR-30b-5p, miR-19a-3p, and miR-130a-3p in serum extracellular vesicles compared with control animals, highlighting their potential role in the development of airway inflammation in this model [31]. In a mouse model of ovalbumin (OVA)-induced asthma, bronchial epithelial cells (BEC) and macrophages display enhanced extracellular vesicle (EV) secretory activity compared with other cell types, as determined by immunohistochemical analysis. These EVs are proposed to stimulate macrophage proliferation and chemotaxis, thereby intensifying the inflammatory response [55]. Dietary consumption of bovine milk may influence pulmonary inflammatory responses to agricultural dust (DE) exposure. Mice receiving the diet with EVs disrupted by sonication (DEV) had an increased macrophage influx into lungs and decreased levels of IL-6, CXCL1, and amphiregulin (AREG) in the BAL compared to those fed the intact EV diet. This may suggest that acute lung inflammatory response elicited by DE may be modulated, at least in part, by the EVs cargo present in the diet. Ex vivo stimulation with DE revealed that macrophages from the EV-fed group produced higher levels of M1-associated proinflammatory cytokines, including TNF-α, IL-12/23, IL-6, while levels of M2-associated protective mediators such as IL-10, and arginase were reduced indicating that milk-derived EVs regulate inflammation through effects on macrophage polarization [56]

Experimental models further support the role of EV-associated miRNAs in pulmonary inflammation. Qiao et al. demonstrated dysregulation of several miRNAs in serum exosomes from rats with pulmonary neutrophilic inflammation induced by zinc oxide nanoparticles, suggesting specific miRNAs as mediators of pulmonary inflammation [31]. Among these, miR-134-5p, miR-207, miR-465-5p, miR-30b-5p, miR-19a-3p, and miR-130a-3p were identified as the most likely contributors to pulmonary inflammation in this model (Table 1; Fig. 2).

**Fig. 3:**
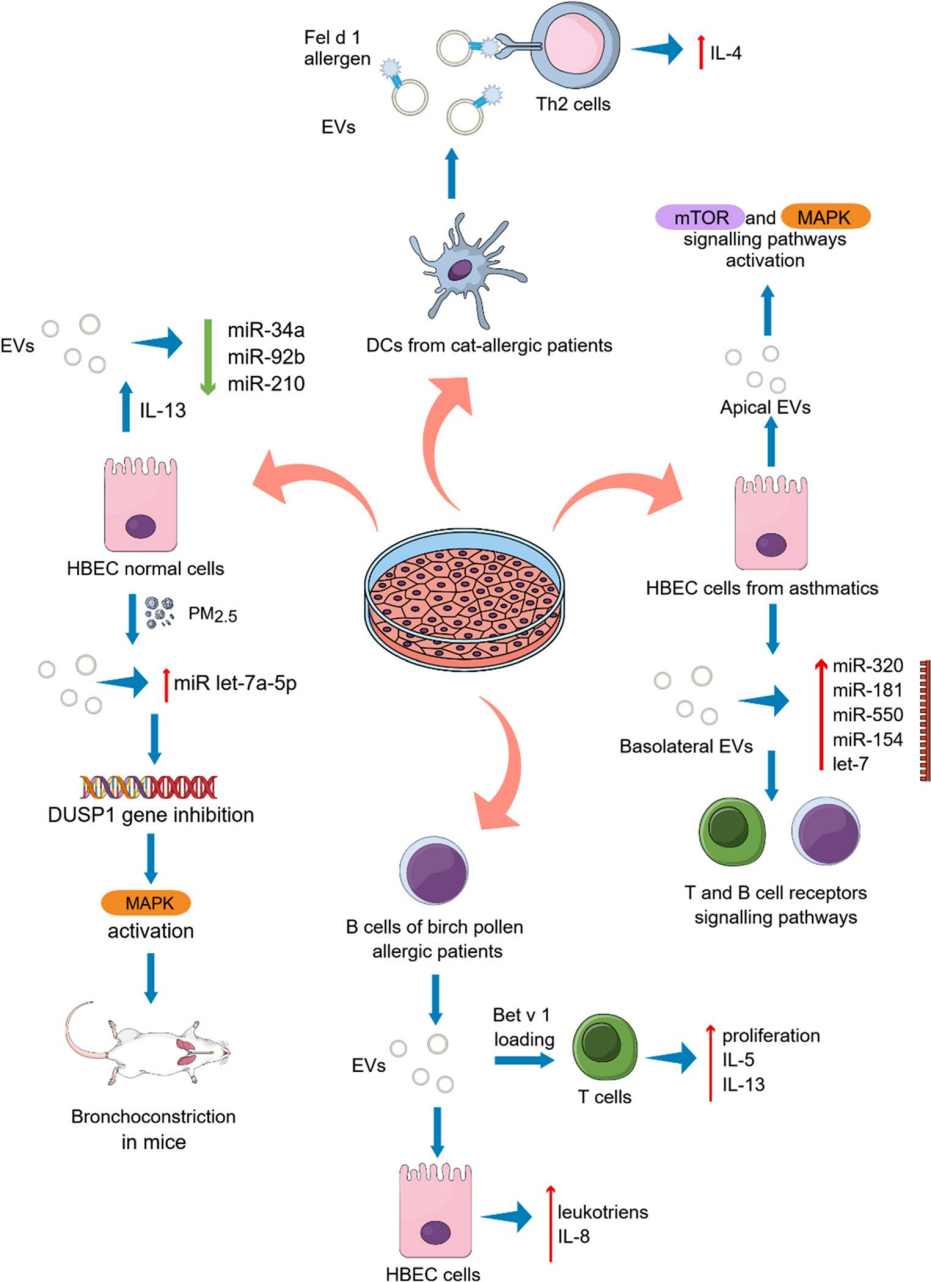
Involvement of EVs isolated from cell cultures in asthma pathogenesis. Human bronchial epithelial cells (HBEC) isolated from asthmatic patients produce apically EVs participating in the mammalian target of rapamycin (mTOR) and mitogen-activated protein kinase (MAPK) signalling pathways while basolaterally secreted EVs are enriched in miRNAs from the miR-320, miR-181, miR-550, let-7, and the miR-154 families involved in the T and B cell receptors signalling pathways [34]. IL-13-treated HBEC secrete EVs with decreased levels of miR-92b, miR‐34a, and miR‐210 associated with airways obstruction probably resulted from the dendritic cell maturation and development of the Th2 response [33]. EVs derived from PM_2.5_- treated HBE cells are enriched in miRNA let-7i-5p which inhibits the DUSP1 gene which is a target in the activation of the MAPK signalling pathway leading to the bronchoconstriction in mice with OVA-induced asthma [35]. B-cells isolated from patients with allergy to birch pollen produce EVs presenting allergen peptides and thus activating specific T cells to proliferate and secrete Th2-associated cytokines, including IL-5 and IL-13 [57]. These EVs carry also leukotriene biosynthesis enzymes and enhance leukotriene and IL-8 production in HBEC [58]. Human DCs from cat-allergic blood donors can present the major cat allergen Fel d 1 to other immune cells and induce Th2-type cytokine production, including IL-4 [59]

Consistent with these findings, elevated levels of miRNA-126 were detected in serum exosomes from asthmatic patients as well as in lung tissues from asthmatic mice, further supporting its involvement in asthma-related disease mechanisms [28] (Table 1; Fig. 1). In vitro studies demonstrate that epithelial cell-derived EVs actively shape immune responses; for example, IL-13-stimulated bronchial epithelial cells release EVs with altered miRNA profiles. An RT-qPCR‐based profiling of the EV‐associated miRNAs revealed decreased levels of miR‐92b, miR‐34a, and miR‐210 in IL-13-treated cells [33] (Table 1; Fig. 3).

Importantly, Bartel et al. determined also the levels of miRNAs in extracellular vesicles from nasal lavages of children with asthma. Lower expression of miR-34a, miR‐92b, and miR‐210 levels compared to healthy controls was connected with airways obstruction (Fig. 1). It was concluded that the above-mentioned miRNAs might play an important role in the dendritic cell maturation and development of the Th2 response in the early stage that leads to the asthma onset. Further complexity in epithelial EVs biology was demonstrated by Schindler et al., who analyzed EVs secreted by bronchial epithelial cells from healthy and asthmatic individuals [34]. Importantly, the biological activity of EVs is highly dependent on their cellular origin and route of secretion. Bronchial epithelial cells release distinct EV populations from apical and basolateral surfaces, which differ in size, molecular cargo, and functional properties (Fig. 3). Those secreted to the basolateral compartment were bigger, more abundant and had higher expression of CD9 and CD81 tetraspanin surface markers when compare to EVs released to the apical cell side. Analysis of miRNAs composition revealed in basolaterally secreted EVs the highest amounts of molecules from the miR-320, miR-181, miR-550, let-7, and the miR-154 families which were involved in the T and B cell receptors signalling pathways (Table 1). Contrary to that, miRNAs enriched in EVs from the apical side of the bronchial epithelial cells were connected with the mammalian target of rapamycin (mTOR) and mitogen-activated protein kinase (MAPK) signalling pathways [34]. This spatial heterogeneity clearly demonstrated the complexity of EV-mediated communication in asthma.

Environmental factors also influence EV-mediated mechanisms in asthma. Exposure to particulate matter (PM2.5) alters the miRNA cargo of epithelial-derived EVs, promoting pro-inflammatory signaling and airway dysfunction, thereby linking environmental factors with molecular mechanisms of asthma [35] (Table 1; Fig. 3).

Clinical studies have reinforced the relevance of EVs in asthma. Paredes et al. found that tetraspanins CD81 and CD63 were expressed in higher amounts in exosomes from BALF of birch pollen-allergic patients than in controls [58]. Owing to their enzymatic cargo involved in leukotriene biosynthesis, these exosomes enhanced leukotriene and IL-8 production in human bronchial epithelial cells, suggesting a direct contribution to asthma pathogenesis. Similarly, Levänen et al. [60] investigated miRNA profiles of exosomes from BALF obtained from asthmatic and healthy individuals. Their analysis revealed, that exosomes from asthmatic patients were enriched with 24 miRNAs compared to those isolated from controls, and these alterations were strongly correlated with lung function expressed as forced expiratory volume (FEV1) values. Furthermore, pathway analysis showed that the altered miRNA were linked to level of cytokines crucial for asthma pathogenesis such as IL-13, IL-10, IL-6, and IL-8, and were associated with the MAPK and JAK-STAT signalling pathways. Importantly, a subset of 16 miRNAs enabled a clear distinction between the asthmatic and healthy groups, suggesting that BALF-derived exosomes may represent a promising tool for asthma diagnostics [60].

In addition to noninvasive sources such as nasal lavage fluid and serum – often referred to as “liquid biopsies” – EVs involved in allergic processes can also originate from diverse immune cell populations. For example, dendritic cells stimulated with thymic stromal lymphopoietin (TSLP) were shown to release exosomes containing the OX40 ligand, a molecule that promotes Th2 differentiation, which represents a key process in asthma pathogenesis [61]. Similarly, neutrophils exposed to lipopolysaccharide (LPS) produce EVs enriched in neutrophil degranulation products, including proteases that promote myocyte proliferation. This process enhances airway smooth muscle proliferation, leading to asthma exacerbation [62]. Notably, eosinophil-derived EVs exert both autocrine and paracrine effects, amplifying inflammatory responses and impairing epithelial repair mechanisms [63]. Further evidence from animal models demonstrates that modulation of EV release influences disease severity [55]. The authors observed increased exosome secretion by bronchial epithelial cells and macrophages compared with other airway cell types (Fig. 2). Moreover, IL-13 modulated both the abundance and molecular composition of epithelial-derived EVs, thereby amplifying allergic inflammation through enhanced monocyte proliferation. Pharmacological inhibition of exosome release using GW4869 reduces airway inflammation and immune cell activation, although excessive inhibition may disrupt physiological homeostasis, indicating a dual role of EVs in maintaining immune balance [55]. As inhaled allergens first come into contact with alveolar macrophages and epithelial cells, their collaboration is crucial for appropriate inflammatory reactions and the preservation of homeostasis. Draijer et al. showed that alveolar macrophages deliver suppressor of cytokine signaling 3 (SOCS3) to alveolar epithelial cells via EVs, thereby contributing to the regulation of lung inflammation by inhibition of JAK-STAT signaling [64]. SOCS3 levels in bronchoalveolar lavage fluid were reduced in asthmatics and allergen-challenged mice. Alveolar macrophage–derived EVs containing SOCS3 inhibited STAT3/STAT6 activation and cytokine expression in epithelial cells, but this effect was lost under allergic inflammation. Administration of SOCS3 liposomes reduced lung inflammation in mice, suggesting SOCS3 delivery may represent a potential therapeutic strategy in asthma.

An equally significant aspect of exosomes’ functions in allergy is their potential role as carriers of aeroallergens. Admyre et al. demonstrated that B cell-derived exosomes possess antigen-presenting capabilities relevant to allergic responses [57] (Fig. 3). They showed that Epstein-Barr virus-transformed B cells isolated from patients with allergy to birch pollen released exosomes expressing high levels of MHC class I and II together with CD40, CD80, and CD86 costimulants, CD19 marker, as well as CD63 and CD81 tetraspanins. When loaded with peptides derived from the major birch pollen allergen Bet v 1, these exosomes induced dose-dependent proliferation of Bet v 1-specific T cells and promoted the secretion of Th2-associated cytokines, including IL-5 and IL-13. This study provided the first evidence that exosomes can present allergen-derived peptides and directly activate allergen-specific T cells, underscoring the contribution of APC-derived EVs to T cell-mediated allergic immune responses and highlighting their potential role in allergy pathophysiology. Vallhov et al. proved that exosomes derived from human dendritic cells (DCs) of cat-allergic blood donors could present the major cat allergen Fel d 1 to the other immune cells, stimulate Th2-like cytokine production (IL-4) in allergic donors [59] (Fig. 3). This finding may have practical implications for the development of novel immunotherapeutic and exosome-based vaccines. Further insights into eosinophil-derived EVs were provided by Canas et al., who compared exosomes isolated from blood eosinophils of asthmatic patients and healthy donors [53]. Exosomes from asthmatic individuals increased ROS and NO production, enhanced eosinophil adhesion to fibronectin, and promoted cell viability, indicating a potential autocrine mechanism that amplifies eosinophil-driven inflammation. Subsequent studies by the same group revealed that these exosomes also induced apoptosis in human small airway epithelial cells and delayed epithelial wound repair [54]. These effects were associated with altered PI3K/AKT and JAK-STAT signaling, as evidenced by reduced phosphorylation of AKT and STAT3 (Fig. 1). In addition, eosinophil-derived exosomes increased the expression of proinflammatory genes, including TNF, CCL26, and POSTN, and promoted bronchial smooth muscle cell proliferation via enhanced MAPK signaling. Collectively, these findings demonstrate the pleiotropic role of eosinophil-derived exosomes in asthma pathogenesis, airway remodeling, and disease exacerbation [54]. Lv et al. used papain-induced asthmatic mouse model to investigate the possible EVs-mediated interaction between the most important populations of innate immune cells in airway tissues specifically, lung M2 macrophages and group 2 innate lymphoid cells (ILC2s) [65]. Immunofluorescence and flow cytometry analyses revealed, that after intratracheal injection of lung M2 macrophage-derived EVs to asthmatic mice, ILC2s were capable of recognizing and interacting with these vesicles. Furthermore, ILC2s can use various endocytic pathways for EVs uptake, allowing them to adapt to changing environmental conditions. Additionally, separated mouse CD4^+^ T cells and macrophages cultured with PKH26-labeled M2 EVs for 24 h also internalized the vesicles, suggesting their indirect ability to promote the activation of ILC2s through macrophages and CD4^+^ T cells. RNA sequencing analysis identified the transcript 4930474H06RiK as a potential mediator of ILC2s activation in allergic airway inflammation, as its expression was highly increased in M2 macrophage-derived EVs from the lung tissue of and asthmatic mice compared with control animals. Functional analyses indicated that this transcript promotes cytokine production and ILC2 proliferation, possibly through downregulation of glycolysis-related molecules such as HK2. These results highlight the role of M2 macrophage-derived EVs in amplifying type 2 immune responses in asthma.

Interestingly, EVs of non-host origin, such as those derived from dietary sources, may also modulate immune responses. For example, bovine milk-derived EVs have been shown to influence macrophage polarization and alter inflammatory responses in the lung, suggesting that exogenous EVs may contribute to the regulation of airway inflammation [56]. Macrophages isolated from mice fed intact milk EVs and stimulated ex vivo with aques extracts of agricultural dust produced higher levels of M1-associated proinflammatory cytokines, including tumor necrosis factor alpha (TNF-α), IL-12/23, and IL-6, while the expression of M2-associated anti-inflammatory mediators such as IL-10 and arginase was reduced (Fig. 2). To sum up, findings demonstrate that EVs are central regulators of asthma pathogenesis, acting through multiple mechanisms including miRNA transfer, immune cell modulation, antigen presentation, and environmental signal integration. Their effects are highly context-dependent, influenced by cellular origin, microenvironment, and disease stage, which underscores both the complexity and therapeutic potential of EV-targeted strategies in asthma.

### Extracellular Vesicles in the Development of Atopic Dermatitis

Atopic dermatitis (AD) is a chronic inflammatory skin disease characterized by barrier disruption, immune dysregulation, and recurrent eczema accompanied by intense pruritus and xerosis. It is one of the most common inflammatory disorders in childhood, affecting up to 20% of the pediatric population, and often precedes the development of other atopic diseases, including asthma, allergic rhinitis, and food allergy. Increasing evidence also suggests that AD has systemic consequences extending beyond the skin [66].

The epidermis is central to AD pathogenesis. Under physiological conditions, its stratified structure and tightly regulated keratinocyte differentiation ensure effective protection against water loss, allergens, irritants, and microorganisms. In AD, however, impaired barrier integrity, abnormal keratinocyte differentiation, and altered immune signaling facilitate allergen penetration and microbial colonization. This is accompanied by a type 2-skewed inflammatory milieu, characterized by increased expression of IL-4, IL-5, IL-13, IL-22, and IL-31, elevated IgE production, eosinophilia, and progressive tissue damage [67].

The regulation of the skin microenvironment relies on a complex interplay between blood, skin cells, immune cells, skin microbiome. Within this context, EVs have emerged as important mediators of this communication. In AD, EVs appear to contribute to disease development through three major mechanisms: microbial induction of skin inflammation, modulation of keratinocyte homeostasis, and regulation of antigen-specific immune responses.

**Fig. 4:**
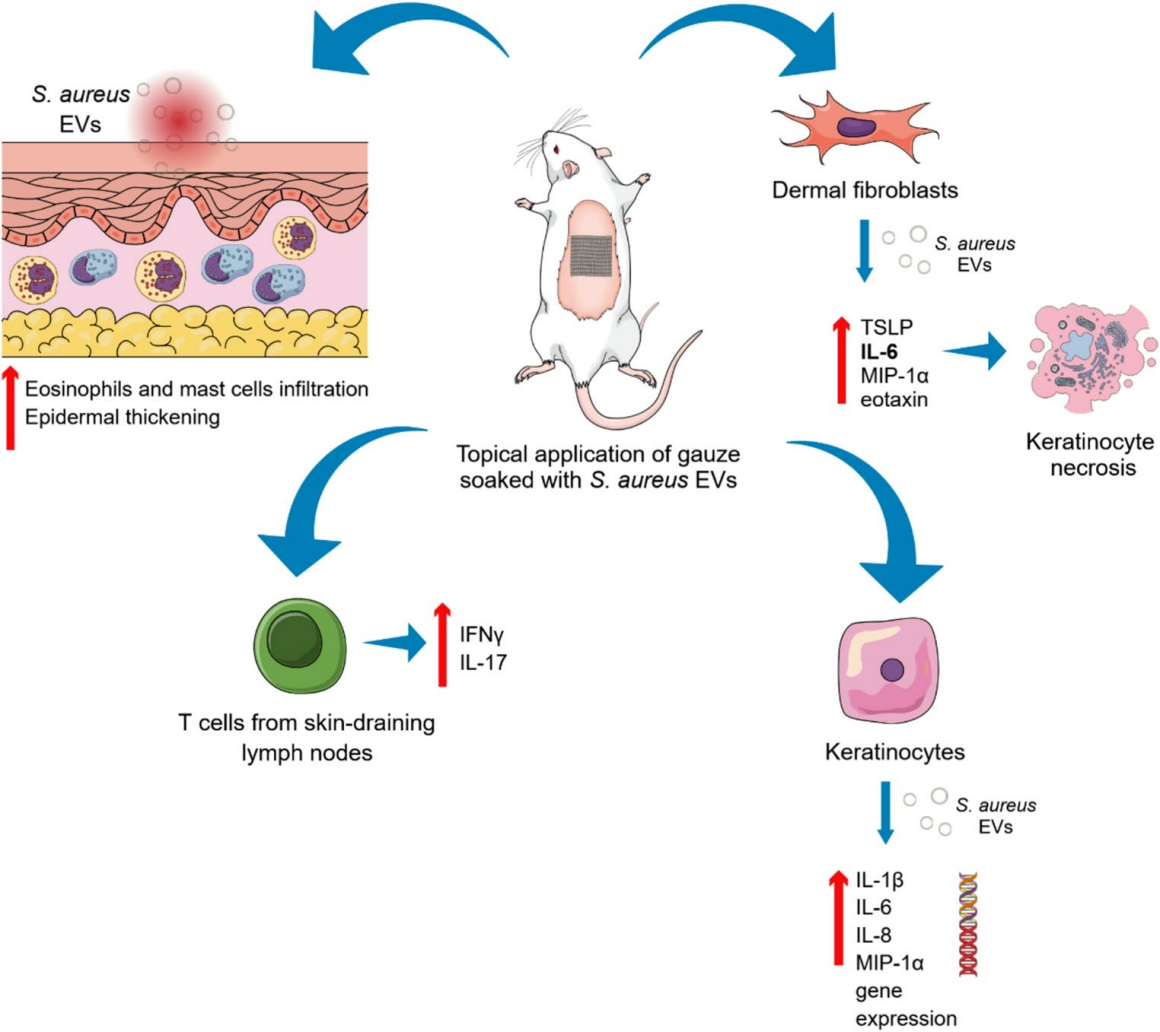
Contribution of *Staphylococcus aureus*–derived EVs to atopic dermatitis pathogenesis. Bacteria that constitute the skin microbiota can release extracellular vesicles (EVs) capable of delivering a variety of bioactive molecules to host cells, thereby initiating inflammatory responses. In a murine model, application of gauze soaked with *Staphylococcus aureus*–derived EVs to tape-stripped skin resulted in epidermal thickening and increased dermal infiltration of mast cells and eosinophils. Additionally, T cells isolated from skin-draining lymph nodes of EV-treated mice exhibited elevated production of IFN-γ and IL-17. In vitro stimulation of primary mouse dermal fibroblasts with *S. aureus* EVs led to enhanced secretion of proinflammatory mediators, including IL-6, TSLP, macrophage inflammatory protein-1α (MIP-1α), and eotaxin [68]. Furthermore, *S. aureus* EVs were shown to contain the virulence factor α-hemolysin, which promotes keratinocyte necrosis by upregulating IL-6 production [69]. These EVs also induced the expression of proinflammatory cytokine genes such as IL-1β, IL-6, IL-8, and macrophage inflammatory protein-1α in keratinocytes. This response was attributed primarily to non-protein EV components, likely peptidoglycan, acting through activation of the TLR2 and NOD2 signaling pathways [70]

Commensal and pathogenic microorganisms residing on the skin surface are capable of releasing EVs that deliver bioactive cargo to host cells, thereby modulating local immune responses. One of the best-characterized examples involves *Staphylococcus aureus*-derived EVs [68]. In a murine model, topical application of gauze soaked with *S. aureus* EVs onto barrier-disrupted skin induced epidermal thickening and dermal infiltration by mast cells and eosinophils. Furthermore, T cells isolated from skin-draining lymph nodes exhibited increased production of IFN-γ and IL-17 (Fig. 4).

Consistent with these in vivo observations, primary mouse dermal fibroblasts stimulated with *S. aureus* EVs in vitro secreted higher levels of IL-6, TSLP, macrophage inflammatory protein-1α (MIP-1α), and eotaxin compared with cells exposed to soluble bacterial fractions. Importantly, patients with AD displayed elevated serum levels of *S. aureus* EV-specific IgE relative to healthy controls, and cutaneous exposure to these EVs increased total serum IgE levels in mice [68]. Subsequent studies identified α-hemolysin as a key pathogenic component of these vesicles [69]. EV-associated α-hemolysin induced keratinocyte necrosis via upregulation of IL-6 and exhibited greater cytotoxicity than its soluble counterpart (Fig. 4). Topical application of these EVs resulted in epidermal hyperplasia, barrier disruption, increased expression of proinflammatory mediators, and the development of AD-like skin inflammation in mice. Further insights were provided by Jun et al. who investigated the role of *S. aureus* EVs in exacerbating AD [70]. They demonstrated in vivo, that *S. aureus* EVs deliver staphylococcal protein A to keratinocytes across all epidermal layers and in vitro induce the expression of pro-inflammatory cytokine genes (IL-1β, IL-6, IL-8, and MIP-1α) (Fig. 4). This effect was caused by non-protein components of EVs, probably peptidoglycan, through activation of TLR2 and NOD2 signalling pathways. Additionally, topical administration of *S. aureus* EVs onto ear lobes of mice with AD-like lesions induced by *Dermatophagoides farinae* extract caused extensive infiltration of inflammatory cells and eczematous dermatitis. These findings underscore that bacterial EVs are not passive byproducts of colonization, but active drivers of epidermal injury and immune activation in AD.

Fungal EVs also contribute to AD pathogenesis. *Malassezia sympodialis*, a common skin commensal associated with AD, releases EVs that stimulate lymphocytes and dendritic cells and promote Th2-related immune responses [71, 72]. Gehrmann et al. demonstrated that this yeast secretes extracellular vesicles (MalaEx), which enhanced IL-4 production in peripheral blood mononuclear cells (PBMC) isolated from both AD patients and healthy individuals (HC) [73]. Blood isolated from these patients was also used for the generation of monocyte-derived dendritic cells (MDDC). Next, MDDC either alone or co-cultured with *M. sympodialis* were used for the isolation of exosomes, DCexo and DCexo Mala, respectively. Interestingly, both types of vesicles contained *M. sympodialis* antigens. Notably, DCexo Mala induced stronger IL-4 and TNF-α production in PBMC from AD patients compared with DCexo, while MalaEx exhibited even more potent immunostimulatory effects. This suggests that MalaEx may present a higher density of immunogenic molecules (pathogen associated molecular patterns (PAMPs)) on their surface than DCexo, contributing to strong activation of innate immune response in AD inflammation [73]. Furthermore, DCs secrete exosomes which transfer functional MHC–peptide complexes, which are already processed and are able to stimulate T cells [74]. Subsequent studies by Rayner et al. revealed that MalaEx carries small RNA molecules ranging from 16 to 22 nucleotides [75]. Interestingly, MalaEx produced under normal skin pH (5.5) or elevated pH (6.1), characteristic of AD skin, did not differ in vesicle number, protein content, size, or small RNA levels. Strong immunostimulatory potential suggests that fungal EVs may amplify host-microbe interactions and sustain cutaneous inflammation. Thus, EV-mediated communication may help explain how skin commensals become pathogenic in the altered microenvironment of AD.

A different perspective on the role of EVs in allergic diseases is presented by researchers studying the properties of EVs produced by probiotics. EVs released by *Lactobacillus plantarum* appear to exert anti-inflammatory effects, reducing IL-6 production by keratinocytes and macrophages in vitro and alleviating skin inflammation, epidermal thickening, and local IL-4 expression in murine models of AD [76]. These findings highlight the dual role of microbiota-derived EVs in AD, acting as either pathogenic or protective modulators of skin inflammation depending on their microbial origin and cargo.

The composition of skin microbiota of AD patients differs significantly from healthy individuals, with *Candida albicans* recognized as one of the most abundant fungal species colonizing skin of AD patients [77]. Its colonisation may lead to the deterioration of AD symptoms, as *C*. *albicans* is recognised by the epidermal keratinocytes that initiate innate immune responses through secretion of chemokines and cytokines, and extracellular vesicles with immunomodulatory properties [78]. In this context, keratinocyte-derived EVs have emerged as key regulators of cutaneous immune responses. Kobiela et al. investigated how an atopic cytokine milieu alters keratinocyte-derived small EVs during exposure to *C. albicans* [79]. AD-associated cytokines (IL-4, IL-13, IL-22, and TSLP), combined with fungal stimulation, modified the glycosylation patterns of sEVs, enhancing their interaction with inhibitory Siglec receptors on dendritic cells. This increased Siglec engagement attenuated innate immune responses and promoted tolerance toward microbial colonization. These changes were driven by altered expression of glycosyltransferases ST6GAL1 and C1GALT1, and indicating that EV remodeling in AD does not merely reflect inflammation, but may actively perpetuate an altered skin microenvironment [79].

Keratinocyte-derived EVs are also involved in epidermal barrier maintenance. Recent studies suggest that they participate in the removal of excess profilaggrin and filaggrin-related products [80]. This is particularly relevant because filaggrin is essential for epidermal structure and immune defense, and its deficiency is strongly associated with AD [81]. Inhibition of EV secretion leads to intracellular accumulation of filaggrin monomers, disruption of cellular junctions, and induction of apoptosis. Remarkably, AD patients exhibited reduced profilaggrin within keratinocytes but increased EVs levels in circulation, suggesting compensatory secretion of filaggrin-rich EVs under conditions of skin barrier stress. Moreover, this mechanism was amplified by colonizing *Staphylococcus aureus* in a TLR2-dependent manner, suggesting that pathogenic bacteria can hijack EVs secretion to weaken antimicrobial responses and support their persistence on AD skin [80].

An additional mechanism linking EVs to AD involves lipid antigen presentation. AD lesions are enriched in CD1a-restricted T cells [82], which recognize lipid antigens presented by Langerhans cells and dermal dendritic cells [83]. Kobiela et al. recently showed that EVs produced by filaggrin-insufficient keratinocytes serve as lipid antigen sources for CD1a-restricted T cells [84]. Although these EVs did not influence classical peptide antigen presentation or IFNγ responses, filaggrin deficiency altered the EV lipid cargo, diminishing the availability of ligands that drive type 1 responses and favoring those that induce type 2 immunity. As a consequence, these altered EVs promoted IL-13 secretion from CD1a-restricted T cells. Lipidomic analysis of EVs revealed decreased long-chain and polyunsaturated fatty acids and elevated amounts of the bulky headgroup sphingolipids characteristic for filaggrin-insufficient keratinocytes-derived EVs. Interestingly, these changes in the lipidomic profile of EVs mirrored analogous lipidomic shifts observed in filaggrin insufficient keratinocytes and the skin samples of AD patients. These shifts were associated with the downregulation of enzymes involved in fatty acid elongation and desaturation, including long-chain-fatty-acid-CoA ligase, elongation of very long-chain fatty acid, and fatty acid desaturase family members [84].

Current evidence indicates that EVs are central regulators of AD pathogenesis rather than secondary markers of inflammation. Their effects are strongly source-dependent: microbial EVs promote or suppress inflammation depending on the organism of origin, whereas keratinocyte-derived EVs regulate both barrier integrity and immune signaling. This source-specific functional heterogeneity is particularly important in AD, where disease progression results from the combined effects of epidermal dysfunction, dysbiosis, and aberrant type 2 immunity. Therefore, EVs may represent not only promising biomarkers of disease activity, but also potential therapeutic targets aimed at restoring barrier function, microbiome balance, and immune homeostasis.

### Extracellular Vesicles in the Development of Food Allergy

The food hypersensitivity, is third the most frequently described allergic disease following asthma and atopic dermatitis [85]. Although often considered a modern health concern, awareness of food allergy dates back to the 5th century BC, when Hippocrates described adverse reactions to cheese caused by “hostile humors”. It is now well-known that these “hostile humors” are in fact IgE antibodies – the central mediators of classical immediate-type food allergy [86]. In recent decades, food allergy (FA) has emerged as a major public health concern, particularly in industrialized and rapidly developing countries. It is estimated that up to 10% of children under the age of three may be affected by this condition. Additionally, approximately 25% of adults have experienced at least one episode of adverse food reaction, which are defined as undesirable and potentially life-threatening responses following the ingestion of or contact with certain foods [87, 88]. In early childhood, cow’s milk, peanuts, and tree nuts dominate as allergens, whereas in adulthood shellfish, fruits, and vegetables are more commonly implicated [89]. Patients with food allergy often have cutaneous sensitisation early in life, with subsequent development of allergy symptoms in the gastrointestinal tract. Clinically, FA encompasses a spectrum of immune phenotypes, ranging from IgE-mediated immediate hypersensitivity to non-IgE-mediated gastrointestinal inflammation driven primarily by cellular immune responses [90]. Neverthless, the mechanisms underlying the breakdown of oral tolerance and the development of allergic sensitization remain incompletely understood.

The intestinal epithelium plays a central role in this process by regulating antigen uptake and immune activation [91]. Under physiological conditions, epithelial tight junctions restrict antigen passage, while controlled transcellular transport allows antigens to be presented to the mucosal immune system in a tolerogenic context [92]. The balance between antigen degradation and presentation, together with environmental factors such as infection, inflammation, or barrier disruption, determines whether immune responses result in tolerance or sensitization [93].

Within this epithelial-immune interface, EVs have emerged as candidate mediators of antigen transfer and immune programming. Intestinal epithelial cells (IECs) release EVs carrying MHC class I and II molecules, enabling them to present antigenic information to immune cells [94]. Notably, basolaterally secreted EVs can cross the epithelial barrier and interact with immune cells in the *lamina propria* or lymphoid tissues [95]. Moreover, molecular profiling of basolteral EVs revealed that, they are enriched in MHC class II and CD63, and also contained CD26 and A33 antigen, suggesting specialized roles in immune communication, potentially through direct interaction with T cells or via their uptake by APCs [95]. Experimental studies further demonstrated that IEC-derived EVs can transport antigen-associated signals to mesenteric lymph nodes and facilitate antigen presentation, supported by the presence of heat shock proteins (hsp71 and hsp90) and adhesion molecules such as MFG-E8 [96]. Under physiological conditions, uptake of food antigens by the mucosal immune system typically induces oral tolerance, a systemic form of antigen-specific immune unresponsiveness. Regulatory T cells (Tregs) are considered central to tolerance induction in response to dietary antigens in a healthy intestine. In parallel, diet-reactive T cells under physiological conditions may undergo apoptosis and be cleared in Peyer’s patches by macrophages, a process associated with IL-10 production that restrains proinflammatory cytokine responses and limits autoimmunity, inflammation, and allergy [97]. At the molecular level, transforming growth factor-β (TGF-β), produced by tolerogenic dendritic cells, is a key driver of Treg differentiation, and its extracellular activation requires integrin αvβ6 [98, 99]. In this context, Chen et al. showed that exposure to protein antigens upregulated αvβ6 expression in IECs and induced release of exosomes carrying both αvβ6 and the protein antigen [100]. These epithelial exosomes were taken up by immature dendritic cells, promoting the development of tolerogenic DCs characterized by TGF-β expression. This highlights a critical role for EVs in maintaining immune homeostasis and preventing inappropriate responses to dietary antigens. In physiological conditions, EV-mediated antigen transfer contributes to oral tolerance. However, this regulatory system is highly context-dependent and can be disrupted. Dysbiosis, epithelial barrier dysfunction, and altered immune signaling may shift EV-mediated communication toward sensitization rather than tolerance. In this context, EVs not only reflect immune imbalance but also may actively contribute to disease development.

Importantly, recent studies have extended this concept by demonstrating that dendritic cell-derived EVs can directly promote pro-allergic immune responses. Tucis et al. showed that EVs from LPS or LPS + OVA-stimulated dendritic cells increased IL-4 production and reduced IFN-γ in naïve T cells from egg-allergic patients, indicating a shift toward Th2 polarization [101]. Notably, these EVs required CD3/CD28 co-stimulation to activate T cells, suggesting that they function as a modulatory “third signal” that amplifies antigen-specific responses. Similarly, Serrano-Santiago et al. demonstrated that EVs derived from dendritic cells of infants with cow’s milk allergy induced proliferation of both Th1 and Th2 cells but selectively enhanced IL-4 production, even in cells from non-allergic donors [102]. These EVs also promoted eosinophil activation, increasing reactive oxygen species production and migration, and exhibited cell-type-specific intracellular localization patterns. Together, these findings indicate that DC-derived EVs can propagate and amplify allergic inflammation by coordinating interactions between lymphocytes and effector cells. Oral tolerance, however, is not determined by epithelial signaling alone. It emerges from the integration of multiple protective factors, including functional Treg networks [103], a diverse and balanced gut microbiota, and intact epithelial barriers. Conversely, dysbiosis may impair tolerance and increase susceptibility to FA, and infants with reduced microbial diversity are at higher risk of sensitization [104]. Additionally, the skin functions as a protective barrier, preventing antigen entry, and maintaining its integrity is essential for reducing the risk of allergic sensitization via cutaneous routes [105]. Alongside these endogenous tolerance mechanisms, early-life exposures may further shape immune trajectories. Breast milk contains immunomodulatory components, including exosomes, that support immune development and may reduce allergy risk [106]. In vitro studies show that milk-derived exosomes suppress proinflammatory cytokines (IL-2, TNF-α, IFN-γ) and increase Treg cells, potentially promoting oral tolerance in infants [107]. A key mechanism likely involves EV-associated miRNAs, whose lipid bilayer protects them from degradation in the infant gut and enables their uptake by intestinal cells [108].

Interestingly, research by Van Herwijnen et al. revealed that some of the most abundant in human and porcine milk miRNAs (such as et-7 family members let-7a, let-7b, let-7f, and miR-148a) were common also for cow and panda milk and were involved in the immune processes. Their evolutionary conservation and incorporation into milk-derived EVs suggest a conserved function in modulating immune and cellular processes in neonates [109]. Furthermore, Kosaka et al. identified miR-181a and miR-17 in human milk exosomes, with miR-181 implicated in B-cell differentiation, further supporting the immunoregulatory capacity of milk EV cargo [36] (Table 1). A significant analysis of breast milk microRNAs and their potential roles in establishing oral tolerance and preventing allergies in infants was provided by Ahlberg et al. [37]. The authors identified 10 the most expressed miRNAs in human breast milk as let-7-5p family, miR-148a-3p, miR-30-5p family, miR-200a-3p + miR-141-3p, miR-22-3p, iR-181-5p family, miR-146b-5p, miR-378a-3p, miR-29-3p family, miR-200b/c-3p and miR-429-3p. The functional analysis indicated that these miRNAs are involved in pathways related to physiological immune processes such as TGF-beta signalling (miR-148a-3p, miR-181-5p, let-7-5p and miR-148a-3p), T cell receptor signalling (let-7-5p, miR-30-5p), Toll-like receptor signalling (miR-29-3p), JAK-STAT signalling (let-7-5p), and Th1 and Th2 cell differentiation crucial in allergy development (miR-29, miR-181-5p). They were also associated with broader regulatory networks such as PI3K-Akt, MAPK, TNF, and FoxO signaling, collectively supporting a plausible contribution of milk-derived miRNAs to immune development and tolerance establishment (Table 1). At the same time, the authors emphasized the need for in vivo studies to better define causality and biological relevance [37].

These observations naturally raise a translational question: are exosomes merely passive messengers between epithelial and immune cells, or can they be engineered into antigen-sensing vehicles suitable for targeted immunotherapy? Initial studies provide evidence for both possibilities. Using an oral tolerance model in C3H/HeN mice, Van Niel et al. found that intraperitoneal injection of OVA-loaded epithelial exosomes did not induce humoral or cellular tolerance to OVA [96]. However, when exosomes were generated in the presence of IFN-γ, they elicited a strong OVA-specific immune response despite lacking classical costimulatory molecules, suggesting that they may serve as vehicles sensing the antigenic content of the gut lumen. In contrast, Chen et al. demonstrated that intravenous administration of antigen/αvβ6-carrying exosomes suppressed antigen-specific Th2 responses in Balb/c mice, supporting the concept that appropriately configured exosomes may exert therapeutic effects in antigen-driven inflammation such as allergy [100].

Further support for engineered EV approaches comes from studies enhancing key tolerogenic signals. Because IL-2 is essential for Treg differentiation, Yu et al. investigated modified exosomes carrying IL-2 and displaying specific antigen on their surface (mExosomes) in experimental FA [110]. BMDCs were incubated with IL-2 and OVA to generate mExosomes, which contained OVA-MHC II complexes as well as IL-2, CD9, CD63, and CD81 molecules. These mExosomes increased expression of Bcl-6, TGF-β, and Foxp3 in OVA-specific CD4⁺ T cells, promoting inducible Treg generation. In vivo, seven days of intraperitoneal mExosome treatment ameliorated FA manifestations, including diarrhea, elevated serum IgE and mast cell protease-1, increased Th2 cytokines in intestinal tissue, and epithelial barrier damage [110]. A complementary strategy was proposed by Zeng et al., who generated IEC-derived exosomes carrying IL-10 and OVA/MHC II complexes (IL10CARs) using MODE-K cells incubated with vasoactive intestinal peptide (VIP) and OVA. These vesicles bound OVA-specific CD4⁺ T cells and promoted their differentiation into antigen-specific type 1 regulatory T cells (Tr1). Intraperitoneal administration of IL10CARs attenuated allergic manifestations in experimental FA, an effect dependent on IL-10 signaling, as treatment with the IL-10 inhibitor AS101 abolished the therapeutic benefit [111]. These findings suggest that EVs can be harnessed for antigen-specific immunotherapy, offering a targeted approach to restoring immune tolerance.

Beyond tolerance induction, EVs may also modulate effector pathways central to FA pathophysiology. Mast cells are key effectors of immediate hypersensitivity, releasing histamine, cytokines, and chemokines following IgE-mediated activation [112]. Kim et al. investigated the therapeutic potential of *Bifidobacterium longum* KACC 91,563 in a mouse model of FA [113]. Daily intragastric administration of the freeze-dried bacteria significantly reduced FA symptoms and intestinal mast cell numbers, without affecting Treg counts or shifting the Th2 response toward Th1. Further analysis showed that EVs secreted by *B. longum* KACC 91,563 induced apoptosis in mast cells in the intestinal lamina propria but did not affect T cells, B cells, or eosinophils. Interestingly, bone marrow–derived mast cells (BMMC) internalized EVs not by random phagocytosis but through a specific receptor-mediated pathway yet to be precisely identified. Moreover, ESBP (one of the main EV proteins) injected intraperitoneally reduced diarrhea and mast cell numbers in FA mice without altering Treg or eosinophil levels. These findings suggest that probiotic-derived EVs may represent a novel therapeutic strategy for allergic diseases targeting mast cells. Mast cells were also the focus of a study by Cho et al. [114]. In the Cow’s milk allergy (CMA) the major allergenic proteins have been identified in the casein group. The authors developed mouse model of casein-mediated food allergy (CIA). Plasma EV concentrations were higher in CIA than in normal mice. These EVs showed increased levels of tetraspanins CD9, CD63 and pro-inflammatory cytokines (IL-6, IL-8, TNF-α). CIA-derived EVs also induced strong degranulation of human and mouse mast cells, associated with increased Lyn kinase levels, suggesting a role for EVs in promoting mast cell activation in CMA. Another CMA model was established with bovine β-lactoglobulin (BLG) and alum, Ma et al. observed elevated Th2 cytokines (IL-4, IL-5, IL-13) and identified marked alterations in serum exosomes, particularly downregulation of cholesterol metabolism-related proteins [115]. These changes coincided with systemic lipid dysregulation, including increased total cholesterol, triglycerides, and LDL-c, hepatic lipid deposition, upregulation of sterol regulatory element binding factor 2 and its downstream gene HMGCR in the liver, and increased expression of responsible for intestinal absorption of sterol lipids Niemann-Pick C1-like protein 1 in the small intestine, suggesting enhanced sterol absorption and reabsorption. Notably, pharmacological inhibition of exosome release using GW4869 alleviated these metabolic disturbances, implicating exosomes as mediators linking CMA to lipid metabolic remodeling [115]. Further studies using the CMA model confirmed that blockade of exosome release alleviated hypersensitivity reactions [116]. GW4869 treatment altered CMA-derived exosome concentration and size distribution and improved clinical signs of anaphylaxis. Serum histamine and BLG-specific IgE levels decreased, and exosome blockade influenced T helper differentiation in splenocytes through transcriptional and posttranscriptional regulation of T-bet and GATA3, without affecting cytokine levels such as IFN-γ and IL-4. Additionally, reduced RORγt expression in splenocytes, together with decreased IL-17 A in intestinal homogenates, suggested that exosomes may also support Th17 differentiation in CMA [116]. Together, these findings expand the role of EVs beyond antigen trafficking, implicating them in systemic immunometabolic regulation during FA.

Finally, EV-mediated immune suppression also extends to non-IgE-mediated hypersensitivity. In delayed-type hypersensitivity model to casein, EVs derived from CD8^+^ suppressor T cells effectivey inhibited the inflammatory responses via transfer of miRNA-150, demonstrating that EVs can mediate immune suppression across different types of food-related immune reactions [38] (Table 1). Notably, EVs from B1 cells were ineffective unless supplemented with miRNA-150, highlighting the critical role of this microRNA. Furthermore, casein-specific T cell–derived EVs from CBA mice were able to suppress inflammatory responses in various strains of mice (CBA, BALB/c, and C57BL/6) demonstrating that the intercellular communication mediated by these suppressive EVs is conserved across different genetic backgrounds [38].

Current evidence therefore indicates that EVs participate in food allergy development at multiple levels of immune regulation. Importantly, their function depends on their cellular and environmental origin, highlighting the need for careful interpretation of experimental data. IEC-derived exosomes can transport antigen-associated signals across the epithelial barrier and shape mucosal immune programming; milk-derived EVs may support tolerance through conserved immunoregulatory miRNAs; engineered exosomes can promote antigen-specific Treg responses and suppress allergic inflammation; and systemic exosome pathways may connect allergic immunity with broader metabolic and inflammatory networks. This complexity positions EVs as both biomarkers of disease activity and promising platforms for the development of antigen-specific therapies in food allergy.

## Therapeutic Potential of Extracellular Vesicles

Extracellular vesicles possess several unique properties that position them as highly promising candidates for the treatment of immune-mediated disorders, including allergies. Firstly, compared to cell-based therapies, EVs offer a cell-free approach that minimizes the risk of transferring oncogenic material or unwanted cellular components, thereby improving their safety profile. Secondly, EVs display remarkable stability under conditions that often limit the clinical applicability of conventional biologics. They can withstand acidic environments, osmotic stress, and variable oxygen levels, which enhances their potential for systemic or mucosal delivery [117]. Importantly, EVs derived from different cellular sources display immunomodulatory properties, enabling them to regulate key processes involved in allergic inflammation. Depending on their origin and cargo, EVs can promote immune tolerance, suppress hypersensitivity reactions, or modulate metabolic and inflammatory pathways associated with disease progression. EVs may not only be used as biomarkers but also as active therapeutic agents. Evidence suggests that EV-based strategies may offer a versatile platform for targeted and personalized interventions in allergic diseases. In the following subsections, we summarize key approaches and experimental studies supporting this concept.

### Therapeutic Potential of EVs in Allergic Asthma

Asthma treatment is usually symptomatic and relies on inhaled corticosteroids and beta-adrenergic agonists. Although effective, both drug classes may cause adverse effects, especially during long-term or high-dose use. Corticosteroids may impair growth in children and contribute to osteoporosis and adrenal suppression in adults [118], while beta-adrenergics, may paradoxically aggravate airway inflammation and induce cardiovascular side effects such as tachycardia and elevated blood pressure [119]. These limitations highlight the need for innovative therapeutic strategies that provide long-term disease control without significant systemic toxicity. In this context, EVs have emerged as promising therapeutic candidates because they can modulate immune responses, deliver bioactive cargo, and promote tolerance in a cell-free format. One of the most compelling concepts in EV-based asthma therapy is the induction of allergen-specific tolerance. Tolerogenic exosomes isolated from BALF of allergen-exposed but immunologically tolerized mice have been shown to suppress allergic sensitization and airway inflammation in murine models. These vesicles reduce allergen-specific IgE and IgG1 production, decrease Th2-associated responses, and attenuate inflammatory cell infiltration in the lungs [120]. Importantly, their effects are not always restricted to the original sensitizing allergen., Research by Prado et al. revealed that tolerogenic exosomes specific for the Ole e 1 allergen not only prevented sensitization to the homologous allergen but also inhibited immune responses against an unrelated pollen protein, Bet v 1 from birch [121], suggesting that tolerogenic EVs may induce a broader mucosal regulatory program. Similar conclusions were reported by Almqvist et al., who demonstrated that serum-derived exosomes from mice orally fed OVA suppressed allergic sensitization when administered intraperitoneally to naïve recipients prior to allergen exposure [122]. Together, these studies provide convergent evidence that tolerogenic exosomes can function as preventive immunotherapy, targeting the sensitization phase rather than only treating established disease.

Major line of investigation focuses on EVs derived from mesenchymal stem cells (MSCs), which are multipotent progenitors isolated from bone marrow, adipose tissue, synovium, skeletal muscle, periosteum, trabecular bone, and even deciduous teeth [123]. The anti-inflammatory properties of MSCs are largely mediated through paracrine mechanisms, and EVs appear to reproduce many of these beneficial effects without the risks associated with whole-cell therapy [124].

Across multiple experimental models, MSC-derived EVs suppress airway inflammation, reduce eosinophil and neutrophil infiltration, decrease mucus hypersecretion, and improve airway hyperresponsiveness. Du et al. showed that exosomes isolated from human bone-marrow derived MSCs caused an increase in the anti-inflammatory cytokines levels IL-10 and TGF-β1 produced by the PBMC from asthmatic patients [125]. Incubation of PBMC with MSCs-derived exosomes also induced the Treg differentiation and enhanced their immunosuppressive activity, supporting their therapeutic relevance in asthma. Further support for the therapeutic potential of MSCs-derived EV comes from studies using induced pluripotent stem cells (iPSC-MSCs). Fang et al. investigated small EVs released by iPSC-MSCs and showed that they suppress group 2 innate lymphoid cells (ILC2s), which are key drivers of allergic airway inflammation [39]. In an IL-33 induced eosinophilic airway inflammation model, intravenous MSC-sEV administration reduced ILC2 levels, inflammatory infiltration, mucus secretion, Th2 cytokines (IL-5, IL-13), and airway hyperresponsiveness. RNA sequencing implicated miR-146a-5p as a potential mediator of these effects, consistent with its known immunoregulatory roles (Table 1). To address practical limitations in EV production yield, Bandeira et al. compared natural MSC-derived EVs with EV-mimetic nanovesicles (NVs) generated by mechanically extruding MSCs through polycarbonate membranes [126]. In a murine OVA asthma model, NVs reduced BAL eosinophils and Th2 cells more effectively than conventional EVs. Both intranasal and intraperitoneal delivery were beneficial, though systemic administration produced a more pronounced reduction in eosinophil infiltration and additionally reduced IL-13 levels in BALF. These findings suggest that EV-mimetic platforms may provide scalable alternatives while retaining, or even enhancing, immunomodulatory efficacy.

Other MSCs-EV studies have focused on defined miRNA cargo. Feng et al. showed that rat adipose-derived MSC (ADSC) exosomes carrying miR-301a-3p inhibited proliferation and migration of platelet-derived growth factor BB-stimulated airway smooth muscle cells, while reducing inflammatory cytokine production (TNF-α, IL-1β, IL-6) through STAT3 targeting [40]. In vivo, miR-301a-3p-enriched exosomes attenuated OVA-induced airway fibrosis and lowered inflammatory mediator levels (IL-1β, IL-6, TNF-α, and MCP-1) in lung tissue (Table 1). Notably, circulating miR-301a-3p was reduced in asthmatic patients, supporting its dual relevance as biomarker and therapeutic candidate. Similarly, Mun et al. demonstrated that intranasal administration of ADSC-derived EVs reduced airway hyperresponsiveness, total inflammatory cells and eosinophils in BALF, and OVA-specific Ig levels, while decreasing IL-4 and increasing Tregs in lung-draining lymph nodes [127]. Interestingly, these effects were more pronounced with EV administration than with whole-cell ADSC therapy, reinforcing the concept that EVs may function as optimized delivery systems for the beneficial paracrine effects of MSCs. A related observation was reported by Yang et al. in a murine allergic rhinitis (AR) model, where intravenous administration of human ADSC-derived EVs reduced nasal symptoms and inflammatory infiltration and lowered serum OVA-specific IgE, IL-4, and IFN-γ while improving the Th1/Th2 balance [128].

Umbilical cord-derived mesenchymal stem cells (MSCs) represent an additional clinically relevant source of therapeutic EVs. EVs derived from these cells effectively attenuate severe, steroid-resistant asthma in experimental models, reducing airway hyperresponsiveness, neutrophilic inflammation, and pro-inflammatory cytokine levels while increasing IL-10 production [129]. These effects are largely mediated through modulation of macrophage polarization, shifting macrophages from a pro-inflammatory M1 phenotype toward an immunoregulatory M2 state, evidenced by decreased expression of iNOS and CD86 and increased Arg1 and CD206 expression. Importantly, depletion of macrophages abolishes the therapeutic benefit, highlighting their central role in EV-mediated protection [129].

The biological activity of MSC-derived EVs can be further enhanced by modifying culture conditions. Hypoxic preconditioning increases the anti-inflammatory and antifibrotic properties of EVs, leading to greater reductions in total inflammatory cells, in particular eosinophilic inflammation, Th2 cytokines, and airway remodelling [41]. In vitro, treatment of TGF-β1-stimulated human lung fibroblasts with EVs decreased the expression of profibrotic markers p-smad2/3, α-smooth muscle actin (α-SMA), and collagen-1, suggesting suppression of fibrogenic pathways responsible for airway remodeling. These effects are at least partly mediated by EV-associated miRNAs, particularly miR-146a-5p, which regulates key signaling pathways involved in inflammation and fibrosis.They further demonstrated that miR-146a-5p could be delivered to lung tissue via EVs, supporting its role in the protection against OVA-induced asthma [41] (Table 1). Beyond macrophage regulation, MSC-derived EVs influence multiple immune cell populations. They inhibit dendritic cell activation, promote regulatory T cell (Treg) expansion, and suppress Th2 responses through IL-10–dependent mechanisms. Peng et al. investigated the immunoregulatory effects of MSC-derived small EVs (MSC-sEVs) on dendritic cells from patients with allergic rhinitis [130]. MSC-sEVs were isolated from iPSC-MSCs and co-cultured with human monocyte-derived DCs to generate sEV-immature DCs (sEV-iDCs) and sEV-mature DCs (sEV-mDCs). MSC-sEV treatment downregulated DC expression of CD40, CD80, CD86, and HLA-DR, inhibiting monocyte differentiation into iDCs. Conversely, MSC-sEVs enhanced the phagocytic capacity of mDCs without altering their maturation marker expression. When sEV-mDCs were co-cultured with CD4⁺ T cells from allergic rhinitis patients, the percentage of IL-13⁺CD4⁺ and CD9⁺CD4⁺ T cells was significantly reduced, indicating suppression of Th2 responses via an IL-10–dependent mechanism. This was accompanied by an increase in Tregs, highlighting the ability of MSC-sEVs to promote tolerance [130]. In parallel, MSC-derived EVs can also target Th17-driven inflammation by inhibiting CD4⁺ T cell polarization via modulation of the JAK2-STAT3 pathway [131]. This broad immunomodulatory activity highlights their ability to regulate both eosinophilic and neutrophilic asthma phenotypes. Biodistribution analysis further revealed that intravenously delivered MSC-sEVs accumulated in multiple organs including the liver, spleen, kidneys, heart, and intestines, with the highest enrichment observed in the lungs, confirming efficient systemic distribution through the circulation [131].

Beyond naturally secreted vesicles, engineered EVs offer an additional level of therapeutic precision. Surface-modified EVs can be designed to target specific immune cell populations and deliver defined regulatory cargo. For example, macrophage-targeted EVs carrying miR-511-3p suppress airway inflammation, reduce eosinophilia and goblet cell hyperplasia, and promote M2 macrophage polarization in experimental asthma [42] (Table 1). Complement component 3 (C3) was identified as a major miR-511-3p target, and its inhibition is notable given the relevance of C3 as a therapeutic target in asthma. Such studies demonstrate that engineered EVs can combine cell-free delivery with selective tissue targeting and mechanistic specificity, making them attractive candidates for future precision therapies.

EV phenotypes may also be shaped by established immunotherapies. Matsuda et al. investigated subcutaneous immunotherapy (SCIT) in an OVA-induced asthma model and observed that SCIT attenuated airway hyperresponsiveness and reduced IL-5 levels in BALF [132]. Notably, serum EVs from SCIT-treated mice exhibited increased CD9 expression and suppressed IL-5 production by ILC2s in vitro. The authors proposed that CD9-enriched EVs may inhibit ILC2 activation indirectly through induction of IL-10–producing macrophages. These findings suggest that classical immunotherapy can “reprogram” circulating EVs toward immunoregulatory profiles and raise the possibility of EV-based approaches as safer and potentially more controllable alternatives to allergen extracts, with lower risk of provoking anaphylaxis [132].

Another emerging area involves microbiota-derived EVs. Vesicles released by commensal or probiotic bacteria can attenuate asthma-related inflammation by modulating epithelial and innate immune signaling. In pathogenic settings, bacterial EVs may transport virulence factors, modulate host immunity, enhance antibiotic resistance, or suppress microbial competitors. This dual capacity to either exacerbate or modulate immune responses underscores the potential of bacterial EVs as both therapeutic targets and delivery vehicles in immune-mediated disorders [133, 134].

Sim et al. investigated the influence of commensal *Micrococcus luteus*- derived EVs (MIEVs) on neutrophilic asthma [43]. It was shown that asthmatic patients had significantly lower levels of MIEV-specific IgG4 in the serum compared to healthy controls. Further studies on LPS-induced mouse asthma model revealed that mice treated intranasally with MIEVs had a decreased number of neutrophils in the BALF, reduced infiltration of the immune cells in the lung tissues, and lower epithelial thickness compared to the control group. Interestingly, fluorescence assays detected these EVs exclusively in the lungs, with no presence observed in the heart, liver, spleen, or kidney. MIEVs also downregulated 7 miRNAs and upregulated 6 miRNAs, inhibiting the production of IL-8 and phosphorylation of p65 in human airway epithelial cells (AEC, cell line A549) stimulated with LPS. These cells were then used to isolate EVs to study their effects on monocytes derived from the blood of asthmatic patients. Among the miRNAs identified, hsa-miR-4517 was most abundant in AEC-derived EVs and played a key role in suppressing IL-1β production and NLRP3 expression in LPS-stimulated monocytes (Table 1). This inhibition of monocyte activity led to the inactivation of type 3 innate lymphoid cells [43]. Such findings are particularly important because they expand the therapeutic EV landscape beyond mammalian cells and highlight the role of host-microbiota communication in airway inflammation. Consistent with these results, Lee et al. reported immunomodulatory extracellular vesicles derived from the commensal *Lactococcus lactis* [135]. In BALB/c mice with OVA-induced asthma, intranasal EV treatment reduced airway hyperresponsiveness, eosinophil counts, mucus production, and Th2 cytokines (IL-5 and IL-13), while increasing IFN-γ levels in BALF. In human peripheral dendritic cells, these EVs induced IL-12p70 via p38 MAPK activation, suggesting a shift toward Th1-supportive immunoregulation. Clinically, EV-specific IgG4 levels were reduced in asthma patients and correlated inversely with serum IL-13 while correlating positively with FEV1, linking EV-associated immune recognition to disease severity and lung function. EVs produced by another probiotic bacterial strain, *Lactobacillus paracasei* (LpEVs), have also shown the therapeutic potential in neutrophilic asthma [136]. Mice with LPS-induced neutrophilic asthma (NA) had a lower proportion of *Lactobacillus* species in gut microbial EVs and patients with NA had decreased levels of LpEV-specific IgG4. LpEVs reduced airway hyperresponsiveness, neutrophil infiltration, and CXCL1 and IL-17 levels in bronchoalveolar lavage fluid, while metabolomic analysis identified bioactive compounds (D-(−)-tagatose, palmitoleic acid, and dodecanoic acid) capable of suppressing c-Jun N-terminal kinase (JNK) phosphorylation in primary airway epithelial cells [136]. Together, these findings suggest that bacterial EVs may serve as immunomodulators targeting non-classical asthma phenotypes.

Finally, EV therapeutics are not limited to mammalian or bacterial sources. Plant-derived EVs, which participate in intercellular communication and defense in plants, have also shown immunomodulatory potential. Kim et al. isolated EVs from *Aster yomena* callus cultures (AYC-EVs) and evaluated them in a murine OVA asthma model [137]. Metabolomic analysis revealed that these vesicles contain 17 major metabolites. AYC-EVs significantly suppressed the expression of IL-10, TNF-α and IL-12p70 by LPS-treated mature DCs, as well as the expression of co-stimulatory molecules CD80, CD86, and MHC antigens. Treatment of LPS-stimulated DCs with AYC-EVs also resulted in increased antigen uptake ability and decreased antigen presenting ability in these cells. Furthermore, AYC-EVs can inhibit the phenotypic and functional maturation of DCs since LPS-treated DCs exposed to AYC-EVs reduced the proliferation of CD4 + and CD8 + T cells, and the secretion of Th1 (IFN-γ, IL-2, TNF-α), Th2 (IL-5), and Th17 (IL-17 A) cytokines. In vivo, oral administration of AYC-EVs prior to OVA challenge significantly reduced airway resistance and cellular infiltration by antigen-presenting cells, eosinophils, and lymphocytes. A marked decrease in inflammatory mediators, including TNF-α, IL-4, IL-5, IL-13, eotaxin, and MUC5AC, in BALF was observed, along with reduced total and OVA-specific IgE levels, comparable to dexamethasone treatment [137]. These results suggest that plant-derived EVs may represent an orally deliverable, non-traditional therapeutic modality capable of broad immune modulation and airway inflammation control.

Current evidence indicates that EVs may be therapeutically useful in asthma through several complementary mechanisms: induction of allergen-specific tolerance, suppression of inflammatory effector pathways, regulation of airway remodeling, and targeted delivery of immunoregulatory molecules. Importantly, their activity is strongly source-dependent, with tolerogenic, MSC-derived, engineered, bacterial, and plant-derived EVs acting through distinct pathways. This diversity is both an opportunity and a challenge. On one hand, it broadens the range of possible therapeutic applications; on the other, it underscores the need for careful standardization of EV source, cargo, dosing, and delivery route.

### Therapeutic Potential of EVs in Atopic Dermatitis

The therapeutic landscape of AD is rapidly expanding; however, durable disease control remains challenging, particularly in patients with severe inflammation, chronic barrier dysfunction, and recurrent microbial dysbiosis. In this context, EV-based interventions are gaining attention because they can simultaneously target key pathogenic axes of AD, namely epidermal barier repair, Th2-driven inflammation, itch pathways, and microbe-host interactions, while offering a cell-free and potentially safer alternative to conventional immunosuppression. The studies summarized below illustrate how EVs derived from skin cells, stem cells, engineered nanovesicle platforms, and commensal microbiota can modulate AD phenotypes in vitro and in vivo.

A first proof-of-concept for barrier-directed EV therapy was provided by Jang et al., who investigated exosomes derived from human neonatal dermal fibroblasts (HDFn-Ex) in AD models induced by 1-chloro-2,4,dinitrobenzene (DNCB) [138]. These vesicles reduce oxidative stress in keratinocytes by suppressing ROS production, restore the expression of barrier-related proteins such as filaggrin, involucrin, and loricrin, and promote hydration through activation of PPARα-dependent pathways. Importantly, these in vitro findings translated into in vivo benefit: SKH-1 hairless mice with DNCB-induced AD treated intraperitoneally with extracellular vesicles exhibited reduced hyperkeratosis, decreased epidermal thickness, and lower transepidermal water loss. Treatment restored PPARα, filaggrin, and HAS1 levels and normalized elevated IgE and IL-4 concentrations. In parallel, HDFn-Ex mitigated DNCB-induced IκBα phosphorylation and TNF-α expression, supporting a combined barrier-protective and anti-inflammatory mode of action. Together, these results identify that neonatal fibroblast-derived exosomes can simultaneously improve barrier function and suppress inflammatory signalling in AD [138].

Another example of exosomes with promising therapeutic potential in AD are human adipose tissue-derived mesenchymal stem cell-derived exosomes (ASC-exosomes). These exosomes reduce disease severity, mast cell infiltration, eosinophilia, and Th2-associated cytokines, demonstrating potent anti-inflammatory effects [139]. Interestingly, ASC-exosomes have also been explored in a clinically relevant setting involving treatment-associated adverse events. Dupilumab, a monoclonal antibody approved for the treatment of moderate to severe atopic dermatitis, asthma, and nasal polyps, is associated with dupilumab facial redness (DFR) in approximately 4–10% of treated patients [140]. Han et al. evaluated a topical ASC-exosome formulation applied once weekly for five weeks in adult AD patients diagnosed with DFR. By week 12, most patients exhibited reduced investigator global assessment and clinical erythema assessment scores, accompanied by decreased erythema index across multiple facial sites. At the molecular level, stratum corneum samples showed reduced IL-1α mRNA expression and increased expression of filaggrin and vascular endothelial growth factor. Additionally, protein expression of filaggrin was also upregulated, while expression of IL-1α and human TSLP were downregulated by the exosome application. It is an important observation, since TSLP triggers Th2 cell inflammation leading to the AD development. Altogether these findings demonstrate that topically applied ASC-exosomes may mitigate inflammation while reinforcing barrier-related programs and angiogenic support, thereby alleviating DFR symptoms [140].

A further potentially effective cell-free therapeutic strategy for treating AD is based on neural stem cell-derived EVs (NSC-EVs), which appear to combine anti-inflammatory and regenerative activities. Lee et al. demonstrated that NSC-EVs significantly reduced the expression of proinflammatory cytokines (TNF-α and IL-6) and chemokines (TARC, RANTES, and MCP-1) in stimulated human keratinocytes and macrophages through inhibition of NF-κB pathway phosphorylation [141]. At the same time, they promoted tissue repair through proteins involved in extracellular matrix remodeling. In a DNCB-induced AD mouse model, topical NSC-EV treatment showed efficacy comparable to tacrolimus, significantly reducing mast cell infiltration and improving skin barrier integrity. Proteomic analysis revealed a dual functional profile of NSC-EVs, combining anti-inflammatory proteins such as S100A8, SERPINA1, and ANXA1 with tissue-regenerative factors including FN1, COL1A1, and HSPG2. These findings further support the concept that EV-based therapies may simultaneously suppress inflammation and promote tissue repair in AD.

An emerging strategy to enhance EV efficacy is to “prime” donor cells to enrich vesicle cargo in immunoregulatory factors. IFN-γ-primed iPSC-derived MSC EVs exhibit enhanced immunoregulatory properties, suppressing Th2 signaling pathways (IL-4R/IL-13R–JAK1/STAT6), reducing mast cell infiltration, and downregulating key mediators, including TSLP and IgE receptors [142]. Furthermore, IFN-γ-iMSC-EVs blocked the pruritus by suppressing IL-31R-STAT signaling, increased expression of genes responsible for skin barrier integrity (Filaggrin, Keratin 1, and Keratin 10), and elevated the levels of lipid synthesis-related proteins (serine palmitoyltransferase, HMG-CoA reductase (HMGCR), ceramide synthase 3, ceramide synthase 4). The same AD model was used in further research by Kim et al. to evaluate the therapeutic potential of IFN-γ-iMSC-EVs [143]. Subcutaneous administration of IFN-γ-iMSC-EVs in AD mice reduced skin thickness, inflammatory and mast cell infiltration, and Th2 cytokine signaling (IL-4, IL-13, IL-31), while improving skin barrier integrity. Treatment also reduced inflammation and pruritus, suppressed JAK1/2 and increased Keratin 1 gene expression in IL-4/13-stimulated keratinocytes. Importantly, these effects were stronger than those observed with baricitinib and clobetasol, drugs commonly used in the treatment of autoimmune and inflammatory diseases. Collectively, these results showed that IFN-γ-iMSC-EVs inhibit Th2-induced immune responses, ameliorate skin inflammation, restore AD-induced skin barrier dysfunction, and abnormal lipid synthesis observed in the course of atopic dermatitis. Collectively, these results showed that IFN-γ-iMSC-EVs inhibit Th2-induced immune responses, ameliorate skin inflammation, restore AD-induced skin barrier dysfunction, and abnormal lipid synthesis observed in the course of atopic dermatitis. Consistent findings from an independent model (an *Aspergillus fumigatus*–induced AD model) confirm that primed EVs restore epidermal homeostasis while attenuating immune activation [144]. The authors revealed that the expression of TSLP, IL-25, and IL-33 in the human keratinocyte HaCaT cells stimulated with IL-4 and IL-13 was decreased after treatment with IFN-γ-iExo, while the expression of keratin 1, keratin 10, desmoglein 1, and ceramide synthase 3 was increased. Additionally, mice with *Aspergillus fumigatus*-induced AD treated with IFN-γ-iExo either subcutaneously either epicutaneously had lower epidermal and dermal thickness, as well as lower number of eosinophils, neutrophils, and lymphocytes in the dorsal skin lesions compared to the AD control mice. Serum level of IgE antibodies was reduced, while clinical scores and transepidermal water loss were improved by the IFN-γ-iExo treatment. Transcriptomic analyses of skin tissue from IFN-γ-iExo-treated mice revealed significant enrichment of pathways involved in skin function (keratinization, formation of the cornified envelope) and T cell immune response (Th1, Th2, and Th17 cell differentiation). Overall, these results reinforce the concept that appropriately conditioned stem cell–derived exosomes can restore barrier function and suppress pathogenic immune activation in AD.

Beyond cell-derived vesicles, EV-mimetic nanovesicles provide a scalable platform for drug delivery to inflamed skin. Recently, considerable evidence has accumulated to show that melatonin, an endogenous hormone produced by the pineal gland, may offer therapeutic benefits in AD [145]. Melatonin-loaded nanovesicles (^Mela^NVs) for example, exhibit stronger anti-inflammatory effects than free melatonin, reducing cytokine production, mast cell infiltration, fibrosis markers, and clinical severity in AD models [146]. In the DNCB-induced AD mouse model, topical administration of ^Mela^NVs in contrast to NVs without melatonin led to the alleviation of erythema, edema, and dryness of skin as well as the reduction of average dermatitis severity scores. Furthermore, in ^Mela^NVs-treated mice the infiltration of mast cells in skin lesions was reduced, similarly to the collagen deposition and fibronectin expression, which indicates the mechanism of the AD symptoms improvement caused by ^Mela^NVs. Additionally, treatment of AD mice with ^Mela^NVs led to a decrease in serum IL-4 and IgE levels, and to an increase in IFN-γ levels. Protein expression analysis also revealed decreased expression of proinflammatory COX-2, TNF-α, and protease-activated receptor-2 (PAR-2) in the skin lesions of ^Mela^NVs-treated mice. These findings support MelaNVs as a high-yield, drug-loading platform that can enhance melatonin efficacy and may be adaptable to other dermatologic inflammatory models [146].

Improving local retention is another major challenge in topical AD therapy, and biomaterial-based EV delivery systems may help address this limitation. Wu et al. created oxidized sodium alginate-carboxymethyl chitosan self-cross-linked hydrogels and used them as a carrier for EVs extracted from the pearl oyster *Pinctada martensii* mucus for determination of their therapeutic potential in AD mouse model [44]. They showed that EV cargo such as miR-100-5p can inhibit inflammasome activation via suppression of FOXO3 and downstream NLRP3 signaling, illustrating how specific molecular components drive therapeutic outcomes (Table 1).

Given the strong link between microbial dysbiosis and AD onset and persistence (highlighted in influential work including Weidinger et al. [147]), commensal microbiota-derived EVs have become attractive candidates for therapeutic development. Zhou et al. investigated the role of EVs isolated from commensal skin bacterium *Staphylococcus epidermidis* (SE-EVs) in the calcipotriene (MC903)-induced AD-like dermatitis model [148]. It was shown that SE-EVs induced the expression of proinflammatory genes (TNFa, IL1β, and IL6) in RAW264.7 cells and the expression of human β-defensin (hBD) 2 and hBD3 in HaCaT keratinocytes. Interestingly, these effects resembled the functions of *S. epidermidis.* Topical administration of SE-EVs to mice with AD-like skin inflammation alleviated the most characteristic symptoms, like skin redness, dryness, thickness, transepidermal water loss and itch. These improvements were corroborated histologically by reduction in hyperkeratosis, epidermal hyperplasia, and inflammatory cell infiltration in ear tissue. In vitro, incubation of the MC903-treated HaCaT cells with SE-EVs resulted in the decreased expression of proinflammatory genes, like TNFa, IL1b, IL6, IL8, and iNOS. It was suggested that these effects were exerted through lipoteichoic acid (LTA), since SE-EVs pretreated with an anti-LTA antibody did not provoke similar changes. Furthermore, SE-EVs increased proliferation and migration of the MC903-treated HaCaT cells indicating that they could promote epidermal cell renewal. MC903-treated HaCaT cells incubated with SE-EVs had also increased expression of human defensins hBD2 and hBD3, which resulted from the interaction between SE-EVs and TLR2. It is worth noting that culture supernatants of HaCaT cells treated with MC903 and incubated with SE-EVs inhibited the growth of *S. aureus*. In a MC903-induced AD mouse model, they reduced CD4^+^ T cells, Gr1^+^ cells, Th2 cytokines, TSLP, and total serum IgE. Additionally, SE-EVs induced IL-17 A^+^ CD8^+^ T-cell accumulation in the epidermis [148]. Collectively, these data support the concept that commensal-derived EVs can simultaneously strengthen antimicrobial defense, promote barrier repair, and suppress Th2 inflammation, offering a microbiome-informed therapeutic strategy for AD.

Finally, EV-based therapies are beginning to show translational potential beyond experimental models. In canine AD, both MSC-derived (MSCs; allogeneic exosomes- cExos) and engineered exosomes (xenogeneic exosomes- hExos, human Expi293F cell-derived exosomes loaded with super-repressor IκB) improve clinical severity, restore barrier function, reduce pro-inflammatory cytokines (IFNγ, IL-2, IL-4, IL-12, IL-13, and IL-31), and enhance anti-inflammatory mediators (IL-10 and TGFβ). Importantly, treatment improved skin microbiome richness without reported adverse effects [149]. These findings provide important proof-of-concept for clinical applicability and support further development of EV-based therapies in allergic skin diseases.

Moreover, available evidence indicates that EV-based interventions in AD can be conceptualized along several complementary therapeutic axes: (i) restoration of barrier and hydration programs, (ii) suppression of epithelial alarmins and Th2 cytokine signaling, (iii) modulation of itch-associated signaling, and (iv) microbiome-informed immune training that enhances antimicrobial defense while reducing pathogenic inflammation. The diversity of EV sources, from fibroblasts and MSCs to engineered nanovesicles and commensal bacteria, also suggests that future therapeutic strategies may be tailored to distinct AD endotypes, combining barrier-repair and immune-reprogramming functions within scalable, cell-free platforms.

### Therapeutic Potential of EVs in Food Allergy

Compared with asthma and atopic dermatitis, EV-based therapeutic strategies in FA are still at an earlier stage of development. Nevertheless, current EV-focused interventions in FA largely converge on two complementary goals: direct elimination or reprogramming of allergen-reactive Th2 cells, and reinforcement of intestinal homeostasis, especially during early life, when immune trajectories and oral tolerance are being established. A representative example of antigen-specific EV therapy involves engineered modified vesicles (mEVs) designed to eliminate allergem-specific Th2 cells [150]. Murine bone marrow-derived DC were used by Zhang et al. to obtain modified EVs carrying antigen-MHC II complexes and pro-apoptotic cargo. These EVs selectively recognize allergen-specific CD4⁺ T cells and induce apoptosis via Fas/FasL signaling. Importantly, depletion of T-cell receptor expression by RNA interference abolished mEV binding to OVA-specific CD4⁺ T cells, confirming the antigen specificity of this interaction. In experimental models (DO11.10 and BALB/c mice), this approach reduces intestinal Th2 cell populations, suppresses allergic responses, and increases regulatory T cell frequencies. These findings highlight the potential of EVs as precision immunotherapeutic tools capable of directly targeting disease-driving immune cells while promoting tolerance.

Growing attention has been directed toward strategies aimed at restoring intestinal barrier integrity and immune homeostasis. The intestinal epithelium plays a critical role in preventing antigen translocation into the *lamina propria*, thereby limiting sensitization. It is now well established that gut microbiota and probiotic bacterial strains contribute to barrier strengthening and immune regulation. Martínez-Ruiz et al. explored this concept by investigating EVs isolated from probiotic and commensal *Escherichia coli* strains in a model of early-life immune and intestinal maturation [151]. EVs isolated from probiotic *E. coli* Nissle 1917 (EcN EVs) and commensal *E. coli* strain EcoR12 (EcoR12 EVs) were orally administered to suckling rats during the first 16 days of life. Although body weight was unaffected, both EV preparations increased spleen mass, presumably reflecting heightened immune activity. Levels of circulating IgA, IgM, and IgG were elevated in EV-treated animals, alongside increased proportions of regulatory T cells, cytotoxic CD8⁺ T cells, and NKT cells. Moreover, analysis of immune-related genes revealed that exposure to microbiota-derived EVs led to the upregulation of TLR7 and TLR2 receptors recognizing bacterial antigens and triggering an immune response. On the other hand, either probiotic or commensal EVs downregulated IL-12 and cyclooxygenase-2 expression in the intestine exhibiting anti-inflammatory effects. Additionally, EcN EVs increased villus width, whereas EcoR12 EVs increased villus length, indicating enhanced intestinal structural maturation [151].

A related and increasingly interesting concept involves plant-derived nanoscale vesicles, which may also support intestinal homeostasis and barrier integrity. Numerous studies indicate that plant-derived particles exhibit anti-inflammatory properties in severe intestinal disorders. In this context, Bruno et al. investigated extracellular vesicles isolated from orange juice (*Citrus sinensis*) and demonstrated that these vesicles remained stable under simulated gastrointestinal conditions and could reach the lower gastrointestinal tract with their cargo preserved [152]. Importantly, they were taken up by intestinal epithelial cells without cytotoxicity and modulated the expression of genes involved in inflammatory pathways, including HMOX1 and ICAM1, as well as genes related to tight junction restoration, such as claudins and occludin.

Current evidence indicates that EV-based interventions in FA operate through two major and complementary mechanisms: targeted modulation of allergen-specific immune responses and restoration of intestinal homeostasis. Importantly, the therapeutic effects of EVs are highly dependent on their origin and cargo composition, whether mammalian, microbial, or plant-derived. This source-dependent functional diversity underscores both the promise and the complexity of EV-based strategies and highlights the need for further studies to optimize their design, validate their safety, and define their translational potential in food allergy.

## Extracellular Vesicles as Candidates for the Diagnosis of Allergic Diseases

Extracellular vesicles are increasingly recognized as valuable sources of diagnostic information, as they reflect the molecular and functional state of their cells of origin. EV-based analyses have already been successfully applied as non-invasive diagnostic tools in kidney diseases, cancer, neurodegenerative disorders, and metabolic diseases such as diabetes. A major advantage of EVs is their accessibility in multiple biofluids, including urine, serum, plasma, saliva, cerebrospinal fluid, and breast milk, enabling repeated and minimally invasive sampling for clinical applications [153]. In the context of allergic diseases EVs offer a particularly attractive platform for biomarker discovery and patient stratification. Further evidence supporting the diagnostic utility of EVs was provided by Keller et al., who demonstrated that exosomes can be efficiently isolated from various body fluids, including amniotic fluid, urine, and saliva of healthy donors [154]. Flow cytometry analysis confirmed the presence of characteristic exosomal surface markers (CD24, CD9, Annexin-1, and Hsp70) and verified their correct membrane orientation. They contained also the exosomal shuttle RNA (esRNA) which was protected from degradation by the exosomal bilayer as it was shown in the experiment with RNase A treatment. RNase could cleave esRNA only after sonication of the exosomes. Furthermore, esRNA from amniotic fluid could be used to determine the sex of the fetus and served as a template for the typing of the CD24 single nucleotide polymorphism (rs52812045) thus indicating the usefulness of RNA isolated from exosomes for diagnostic purposes, particularly when cellular material is scarce.

In allergic diseases, EVs provide insights into both inflammatory activity and barrier dysfunction. Proteomic analyses of EVs isolated from nasal lavage fluid have revealed disease-specific signatures in patients with asthma and chronic rhinosinusitis (CRS) [155], including increased levels of proteins associated with epithelial damage and inflammation, and decreased levels of antimicrobial and barrier-related proteins. For example, exosomes from individuals with both asthma and CRS showed reduced levels of S100 proteins (S100A8, S100A9, S100A12) and cathepsin G. These changes may be a sign of elevated predisposition to bacterial and fungal invasion since S100 proteins are involved in antifungal and antibacterial activity, regulation of leukocyte adhesion and migration, and promotion of cytokine and chemokine production, as well as the induction of pro-inflammatory responses in monocytes. Furthermore, in the asthma-only group exosomes had decreased level of two proteins responsible for the barrier function of the skin: filaggrin and hornerin, and three immunoglobulin-related proteins. These alterations may contribute to increased vulnerability to infections and play a significant role in disease progression [155].

In parallel, Fang et al. examined plasma-derived EVs in patients with allergic rhinitis (AR) and found that levels of the major house dust mite allergen Der p 1 were significantly elevated on EVs from both mild and moderate–severe AR patients compared to healthy controls, with expression correlating closely with disease severity [156]. These EVs also expressed a high level of HLA-DR molecule indicating that the circulating HLA-DR⁺ EVs observed in AR patients may be shed by APCs and probably participate in the activation of T cells, especially CD4 + T cells mimicking antigen presentation of APCs in the pathogenesis of AR. Additionally, AR-EVs were taken up by CD4 + T cells and increased the levels of Th2 cells after incubation with purified CD4 + T cells. This work advances understanding of antigen presentation in the allergic responses of AR patients and points to potential targets for novel diagnostic and therapeutic strategies.

Additional evidence supproting the utility of plasma EVs as biomarkers was provided by Wagner et al., who characterized EVs isolated from the plasma of patients with rhinoconjunctivitis [157]. Increased EV concentration and altered cytokine profiles, including elevated Th2-associated mediators and chemokines (TNF-a, IL-4, IL-5, IL-6, IL-17 F, CCL2, and CCL17), have been observed in allergic patients compared to healthy controls. These patterns may enable discrimination between allergic and non-allergic individuals and provide a basis for disease stratification and monitoring of therapeutic responses. EVs ability to integrate information on immune activation, barrier integrity, and allergen exposure offers a multidimensional view of disease processes. However, their clinical implementation requires further standardization of isolation methods, characterization protocols, and validation in large patient cohorts. Given the multifaceted nature of EV cargo and origin, the following sections provide an integrated overview of their diagnostic applications across nucleic acid, proteomic, lipidomic, cellular, and microbiome-derived signatures in allergic diseases.

### EV-associated Nucleic Acids as Diagnostic Markers in Allergic Diseases

EV-associated microRNAs have emerged as promising non-invasive biomarkers in allergic airway diseases, as they reflect both local inflammatory processes and systemic immune status. In asthma, distinct EV miRNA signatures have been linked to disease severity and inflammatory phenotypes. For example, elevated levels of miR-122-5p in plasma- and sputum-derived EVs have been associated with severe asthma and proposed as markers for differentiating neutrophilic and eosinophilic endotypes (Fig. 1) [29]. The diagnostic relevance of EV-associated miRNAs was further further supported by studies demonstrating dynamic remodeling of EV cargo during allergic inflammation [32] (Table 1). In a house dust mite-induced asthma model (HDM), numerous miRNAs were differentially expressed in bronchoalveolar lavage (BAL)-derived EVs, with many showing inverse expression patterns compared to control lung tissue. This suggests selective packaging and export of regulatory miRNAs into EVs rather than passive release. Functional analyses linked these miRNAs to pathways involved in T helper cell differentiation, granulocyte adhesion and diapedesis, leukocyte trafficking, and airway inflammation, with specific candidates such as miR-346 implicated in the regulation of Th2 cytokines, including IL-13. Importantly, pharmacological inhibition of EV release using GW4869 reduced BAL EV levels, Th2 cytokine production, and eosinophilic infiltration, supporting both mechanistic and biomarker relevance of EV-associated miRNAs. Circulating EV miRNAs also show potential for patient stratification [30, 158] Sundar et al. profiled plasma-derived EV miRNAs from nonsmokers, smokers, and patients with COPD to evalute their utility as circulating biomarkers [158]. The RNA-seq analysis revealed up-regulated miR-22-3p, miR-99a-5p, miR-151a-5p, miR-320b, miR-320d, and down-regulated miR-335-5p, miR-628-3p, miR-887-5p and miR-937-3p in COPD exosomes compared to smokers and non-smokers. 11 of up-regulated miRNAs were validated by using exosomes isolated from human bronchial epithelial cells (BEAS-2B) treated with the cigarette smoke extract (CSE). 8 out of them were significantly increased: let-7a-5p, let-7i-5p, miR-29a-3p, miR-99a-5p, miR-100-5p, miR-151b, miR-375, miR-486-5p, and connected with smoking-related carcinogenesis, COPD, and pulmonary fibrosis, as it was shown in different studies. On the other hand, exosomes from CSE-treated human monocyte cells (U937) had lower expression of 4 out of 11 mi RNAs compared to control, indicating that CSE-induced cellular stress led to the changes of exosomal miRNA content depending on the cell type. tRNAs and piRNAs were also differentially expressed in serum exosomes isolated from patients from analysed groups. Pairwise comparison showed differences in expression of tRNA^Lys^, tRNA^Gly^, tRNA^Tyr^, tRNA^Glu^ and tRNA as well as piR-012753 and piR-020813 in non-smokers vs. COPD. In smokers vs. COPD observed differences were related to the levels of tRNA^Gly^, tRNA^Tyr^, tRNA^Leu^, and tRNA^Met^ as well as piR-004153, piR-020813, piR-020450, and piR-016735. Taken together, this study presents a promising approach for discovering novel circulating EV biomarkers in human plasma, which may contribute to the development of innovative diagnostic, prognostic, and treatment methods of chronic inflammatory lung diseases such as COPD. Beyond miRNAs, other small non-coding RNAs, including tRNAs and piRNAs, were also differentially expressed in circulating EVs across study groups. These findings underscore a broader principle: EV-associated nucleic acids sensitively reflect disease-specific inflammatory and environmental conditions. In allergic diseases, distinct miRNA profiles have been associated with asthma severity, T2-high atopic phenotypes, and systemic inflammatory states such as obesity-related inflammation [30] (Table 1). EV-associated nucleic acids represent a highly informative class of biomarkers. However, their clinical implementation will require careful standardization of EV isolation methods, source selection, and analytical workflows, as well as validation in large and well-characterized patient cohorts.

### Lipidomic and Proteomic Profiling of EVs in Allergic Airway Diseases

Beyond nucleic acids, EV-associated lipids provide another rich layer of diagnostic information. In asthma, bronchoalveolar lavage (BAL)-derived EVs display quantitative and qualitative alterations that correlate with disease activity [159]. Increased EV abundance has been associated with blood eosinophilia and serum IgE levels, linking EV release to type 2 inflammation. At the same time, changes in EV surface markers, including increased HLA-DR and ICAM-1 expression, suggest enhanced immune activation within the airway compartment. Lipidomic analysis further demonstrated that the lipid composition of BAL EVs varied not only between healthy (H) and asthmatic (A) subjects but also depending on exposure to second-hand smoke (SHS). Sphingomyelin, an important ingredient of the plasma membrane, was present in higher amounts in the BAL EVs from asthmatics exposed to SHS (AS) compared to SHS-exposed healthy subjects (HS). Conversely, the level of phosphatidylglycerol 34:2 was significantly reduced in both SHS-exposed study groups, and in asthmatic patients, potentially indicating altered surfactant metabolism contributing to impaired lung function. Additionally, ceramide levels were reduced in BAL EVs from both SHS-exposed asthmatics and healthy subjects. Therefore, the authors suggested the involvement of ceramide-phosphates in the anti-inflammatory processes, which are thought to be impaired due to SHS exposure [159]. Importantly, these findings support the concept that specific lipid species are selectively packaged into EVs and participate in intercellular communication within inflamed airways.

Proteomic approaches have also enabled discrimination between asthma and other chronic pulmonary diseases. Comparative analyses of BAL-derived EVs have demonstrated disease-specific protein signatures, with asthma-associated EVs enriched in proteins linked to immune regulation and surfactant homeostasis (Serpin A6 and apolipoprotein A4), whereas other conditions, such as cystic fibrosis, exhibit profiles dominated by oxidative stress and neutrophilic inflammation markers (LCN2, S100A12, SOD2, and GPX3) [160]. This highlights the potential of EV proteomics for differential diagnosis across clinically overlapping respiratory disorders.

In asthma, serum EV proteomics has also identified candidate biomarkers associated with eosinophilic inflammation [161]. Proteins such as galectin-10, eosinophil peroxidase (EPO), and major basic protein (MBP1) show strong associations with blood eosinophilia, exhaled nitric oxide, mucus production, and airway remodeling. Furthermore, it was shown that these potential biomarkers interacted with extracellular signal- regulated kinase, Jun amino terminal kinase, and phosphoinositide 3-kinase as core proteins and were associated with BA-associated genes, such as IL-4 receptor and IL-13 genes indicating their great potential in the development of non-invasive bronchial asthma (BA) diagnostic tools. Notably, higher levels of Gal10, EPO, and MBP1 in EVs isolated from BA patients compared to HC were associated with higher blood eosinophil counts and fractional exhaled NO levels. These correlations were even more robust with mucus production and bronchial wall thickening than with blood eosinophil counts alone. Notably, the levels of Gal10 had stronger inverse correlation with FEV1 values then blood eosinophil counts and prevails over the blood eosinophil counts as a BM for detecting airway obstruction. Expression of Gal10 was also the highest in the peribronchial tissues of BA patients. BA is often accompanied with the chronic rhinosinusitis with nasal polyps (CRSwNP). It was found, that all BMs identified in EVs isolated from EA patients were also upregulated in EVs from CRSwNP patients. These molecules reflected the protein profiles observed in tissues, with the galectin signaling pathway emerging as the most relevant, and highlighted Gal10 in EVs as a potential BM. Furthermore, the authors showed that during eosinophil extracellular trapped cell death, Gal10 levels in the EVs were increased, and it was correlated with the Lund-Mackay score used for evaluation of the degree of sinus opacification in chronic rhinosinusitis (CRS) [161]. The presence of similar EV protein signatures in related conditions, such as chronic rhinosinusitis with nasal polyps, further supports their relevance as shared biomarkers of type 2 inflammatory diseases. Proteomic alterations in EVs are also evident in atopic dermatitis, where plasma EV cargo differs markedly from that of healthy individuals [162]. KEGG pathways selected on the basis of obtained data included mainly platelet activation, leukocyte transendothelial migration, complement and coagulation cascades, phagosome, pathogenic *Escherichia coli* infection, Rap1 signaling pathway. The authors suggested that increased platelet activation might play a role in the development of AD as this process could enhance stimulation and responsiveness to *Staphylococcus aureus* thereby influencing the onset of AD. Therefore, one of the proteins involved in platelet activation, namely SLP-76 tyrosine phosphoprotein (SLP76) (also called lymphocyte cytosolic protein 2), which was upregulated in EVs of AD patients, was chosen as a focus of the authors’ interest. SLP76 plays a central role in TCR signaling and serves as an essential component for regulating FcεRI-induced IL-4 production in basophils. The IL-4 released by basophils, in turn, contributes to the initiation of Th2-type immune responses and AD development. These findings suggest that SLP67 protein might be considered a prospective diagnostic or therapeutic target [162].

### EVs as Indicators of Immune Cell Activation

Extracellular vesicles can serve as circulating indicators of immune cell activation, as their membrane composition reflects the phenotype and activation status of their cells of origin. This property provides a unique opportunity to monitor immune polarization in a minimally invasive manner. Proteomic profiling of EV membrane proteins has demonstrated that specific EV subsets correspond to defined T cell activation states [163]. In particular, HLA-DR has been identified as a marker of Th1/Tc1-associated EVs, and increased levels of CD3⁺HLA-DR⁺ EVs have been observed across inflammatory conditions, including atopic dermatitis. These findings suggest that EV profiling can capture dynamic changes in immune cell activation in vivo and may complement conventional cellular and cytokine-based assays. In the context of allergic diseases, such approaches could enable more precise monitoring of immune polarization and disease activity, supporting both patient stratification and therapeutic evaluation.

### Microbial EVs and Metagenomic Diagnostics

An emerging and particularly intriguing diagnostic avenue involves microbial EVs, which provide insight into host-microbiome interactions in allergic diseases. Samra et al. demonstrated that urine-derived EVs carry disease-specific microbial signatures in children with chronic rhinitis (CR), allergic rhinitis (AR), and atopic asthma (AS) [164]. The DNA extracted from urine-derived EVs underwent 16 S rDNA sequencing followed by taxonomic classification. It was shown that *Actinobacteria* phylum was more abundant in the AS and CR compared to the control group with a prevalence of the members of the *Micrococcaceae* family. Additionally, the members of the *Propionibacteraceae* and *Corynebacteraceae* families were more abundant in the CR group. Another phylum significantly enriched in the EVs isolated from all disease groups was Proteobacteria. In the AS, *Sphingomonadaceae* and the genera *Sphingomonadaceae* and *Comamonadacea*e were significantly overrepresented compared to the controls. On the other hand, the genus *Methylobacteriaceae* from the *Methylobacteriaceae* family was more abundant in all disease groups. Furthermore, the genus *Enhydrobacter* from the *Moraxellaceae* family was detected in higher amounts in the CR compared to the control group. Although this study had some limitations, the results confirmed that urine EVs exhibit distinct microbial profiles based on the allergic condition, including chronic rhinitis, allergic rhinitis, and atopic asthma in children [164]. Given the stability of EVs and the ease of urine collection, this approach is particularly attractive for pediatric diagnostics.

Similarly, serum EV metagenomic profiling by Lee et al. revealed distinct microbiome signatures in asthma patients compared with healthy controls, with specific taxa associated with inflammatory endotypes, including eosinophilic and non-eosinophilic phenotypes [165]. It was shown, that at the phylum level, *Bacteroidetes* was more abundant in asthmatics, while *Actinobacter*,* Verrucomicrobia*, and *Cyanobacteria* were more abundant in healthy controls. At the genus level, 24 bacterial genera were differentially abundant between individuals with asthma and healthy controls. Interestingly, *Escherichia*/*Shigella* bacteria was more abundant in serum of patients with eosinophilic asthma compared to non-eosinophilic asthma patients. Study subjects were also classified according to the granulocytic cell count of their sputum into four inflammatory phenotypes: eosinophilic, neutrophilic, paucigranulocytic, and mixed granulocytic asthma. Only in the last group one phylum, namely, *Comamonas* demonstrated marked prevalence. Taking into account corticosteroid use, no signifcant diferences were observed at the phylum level. This concept has also been extended to atopic dermatitis, where serum microbial EV profiles show strong discriminatory potential between patients and healthy individuals [166]. Using 16 S rDNA metagenomic analysis using next-generation sequencing they found that AD patients exhibited reduced abundance of phylum *Proteobacteria* and *Cyanobacteria*, and increased levels of *Verrucomicrobia*, *Actinobacteria*, and *Firmicutes*. At the family level, *Enterobacteriaceae* and *Enterococcaceae* were dominant in the AD patients, while *Moraxellaceae* and *Pseudomonadaceae* were dominant in the healthy controls. The key biomarkers were chosen to calculate AD diagnostic model on the basis of the highest Linear Discriminant Analysis score. At the genus-level, it was *Acinetobacter*, and *Pseudomonas* which were increased in the control group, and *Escherichia-Shigella*, *Enterococcus*, *Alistipes*, *Klebsiella*, *Veillonella*, *Bifidobacterium*, and *Akkermansia*, which were decreased in the control group. EV-based metagenomic markers, which can be assessed non-invasively, may aid in diagnosing and predicting the risk of AD development. Together, these studies highlight microbial EVs as accessible biomarkers reflecting host-microbiome interactions relevant to allergic disease pathogenesis.

An additional layer of diagnostic information is provided by host immune responses to microbial EVs [167]. Elevated serum levels of antibodies against EVs derived from common environmental bacteria (*Staphylococcus aureus*, *Acinetobacter baumannii*, *Enterobacter cloacae*, and *Pseudomonas aeruginosa)* have been observed in patients with asthma and other lung diseases, suggesting that immune recognition of microbial vesicles may reflect disease severity and environmental exposure. Microbial EVs represent a novel class of biomarkers that integrate microbiome composition with host immune responses. Their accessibility in biofluids and their stability make them attractive candidates for non-invasive diagnostics, particularly for identifying microbiome-associated disease endotypes in allergic disorders.

## Challenges in EV research

### Method-related Constraints

EVs exhibit significant diversity, stemming from their varied origins, components, and functions, which results in a complex heterogeneity that influences their distinct roles in intercellular communication. This complexity and diversity of EVs contributes to several methodological challenges, as well as the limitations of current technologies. A major technical challenge in EV research remains the balance between isolation efficiency and sample purity. Commonly used techniques including ultracentrifugation, ultrafiltration, and polymer-based precipitation, often co-isolate non-vesicular components, including lipoproteins and protein aggregates. These contaminants can significantly interfere with downstream analyses and lead to misinterpretation of EV composition and function [168]. The separation of EVs is complicated by the fact that lipoproteins and other biological contaminants possess similar physicochemical characteristics. This overlap can result in incorrect interpretations of the composition and function of EVs. To address these limitations, a combination of several insulation methods can be used. For example, Zhang et al. applied a three-step protocol consisting of polyethylene glycol (PEG) precipitation, iohexol density gradient centrifugation, and size-exclusion chromatography, achieving high-purity EV isolation with a recovery rate of approximately 71% and minimal protein contamination [169]. Jimenez et al. compared the proteomic profile of serum-derived EVs purified using four different purification methods: ExoQuick ™, Total Isolation Kit Life Technologies Ultracentrifugation, and Ultrafiltration. It was shown that depending on purification protocol, EVs purified from human serum sample deliver different recovery rates, size distributions, and proteomic profiles [170]. The variability in results from isolation methods across different studies underscores the importance of establishing standardized protocols.

Quantification of EVs also remains challenging. Techniques such as Nanoparticle Tracking Analysis and measuring protein content, frequently yield inconsistent outcomes because of sample variability and contamination [171]. Furthermore, the small amount of molecular cargo carried by EVs poses a significant challenge for detection technologies, requiring methods with high sensitivity and scalability to ensure accurate analysis. Recent technological advances aim to overcome these limitations. Single-EV RNA sequencing enables the detection of the diverse and complex RNA species contained within individual EVs, facilitating the correlation of RNA content with specific EV characteristics and allowing detection of low abundance RNA species [172]. Utilizing High-Resolution Flow Cytometry with advanced spectral imaging and multilaser systems facilitates the simultaneous multiplex analysis of surface markers on EVs, including proteins, lipids, and carbohydrates [173]. Furthermore, Atomic Force Microscopy and Cryo-Electron Microscopy provide the capability to visualize intricate structural details of EVs, such as the organization of proteins, lipids, and other biomolecules within their membranes and interiors [174].

### Challenges Connected with Regulatory Barriers and Clinical Scalability of EV-based Diagnostics and Therapeutics

EV-based diagnostics hold immense promise for non-invasive disease detection and personalized medicine. However, their clinical translation faces significant regulatory and scalability challenges [175]. The regulatory frameworks for EV-based diagnostics vary significantly across regions, including the United States, European Union, and Asia. This lack of harmonization complicates global clinical adoption. Challenges include quality control, safety evaluation, and efficacy demonstration, which are critical for regulatory approval [176]. EVs are inherently heterogeneous in size, origin, and cargo, making it challenging to establish consistent benchmarks for clinical use. This heterogeneity complicates regulatory compliance and product characterization. Efforts like EV-TRACK and MISEV guidelines aim to address these gaps, but further alignment among regulatory bodies is needed [2, 177]. Additionally, large-scale clinical trials and robust validation studies are necessary to demonstrate the efficacy and safety of EV-based diagnostics [178, 179].

One of the major challenges is the low yield and efficiency of EV isolation, limiting their scalability for widespread clinical use. Advanced technologies like microfluidics, nanotechnology, and pH-responsive magnetic beads, show promise for improving scalability and purity but require further validation [180, 181]. Moreover, for clinical translation, establishing Good Manufacturing Practice (GMP)-compliant protocols is essential to ensure standardized production of therapeutic-grade EVs through controlled isolation, purification, and quality control. Emerging Microfluidics-based methods and kit-based isolation approaches may support scalable and consistent production. Because EVs surface markers, size, and heterogeneity affect their activity, multiparametric quality control—including proteome profiling, small RNA sequencing, and functional assays—is required to ensure their safety and efficacy [182, 183]. Another important obstacle facing the clinical EVs utilization is the expense and technical demands of current EV isolation and detection methods that limit their use in routine diagnostics. Developing simpler, more affordable platforms is essential to enable widespread adoption of EV-based tests. Also, introduction of new approaches, such as AI-driven analytics and machine learning, have potential to improve scalability and quality control of EV-based processes, but they still need further refinement and validation [184].

Progress in this field will depend on coordinated efforts between academia, industry, and regulatory agencies. Researchers contribute to the development of standardized methods for EV isolation, characterization, and biomarker identification, while industry stakeholders provide expertise in large-scale manufacturing, process optimization, and commercialization strategies. At the same time, engagement with regulatory agencies helps define clear guidelines for quality control, safety, and clinical validation. Such coordinated efforts can facilitate the establishment of harmonized standards, improve reproducibility across laboratories, and accelerate the translation of EV-based diagnostics and therapeutics from experimental settings into clinical practice [179].

## Summary and Concluding Remarks

Extracellular vesicles have emerged as central mediators of intercellular communication at the interface between barrier tissues, the immune system, and the microbiome. Accumulating evidence reviewed in this work demonstrates that EVs are not merely passive carriers of molecular cargo, but active regulators of immune homeostasis whose dysregulation contributes to the initiation, propagation, and chronicity of allergic diseases. By integrating findings across asthma, atopic dermatitis, and food allergy, this work highlights EVs as key molecular hubs linking environmental exposure, barrier dysfunction, and immune dysregulation. Despite distinct clinical manifestations, these diseases share common EV-driven mechanisms, EVs shape early disease development by modulating immune polarization and barrier integrity. In asthma, EVs derived from airway epithelial cells, immune cells, as well as those influenced by environmental factors, carry microRNAs, proteins, and lipids that promote Th2- and Th17-driven inflammation, and airway remodeling. In atopic dermatitis, both microbial and keratinocyte-derived EVs modulate epidermal differentiation, lipid metabolism, and immune activation, linking barrier dysfunction with Th2-skewed inflammation and dysbiosis. In food allergy, EVs regulate antigen transport across the intestinal epithelium and influence the balance between tolerance and sensitization, particularly during early life. Together, these observations indicate that EVs function as integrative platforms coordinating local and systemic responses across different allergic diseases.

EVs also represent a rapidly advancing therapeutic platform. Their cell-free nature, high stability, and intrinsic targeting capabilities offer advantages over conventional cell-based therapies. EVs derived from immune cells, mesenchymal stem cells, epithelial cells, microbiota, and engineered systems have been shown to suppress inflammation, restore barrier function, and promote immune tolerance. Importantly, EVs can be engineered to deliver defined cargo, including microRNAs, cytokines, and antigens, enabling antigen-specific and pathway-targeted interventions. The effectiveness of these approaches across multiple experimental models, including steroid-resistant and non-classical inflammatory phenotypes, highlights their translational potential.

In parallel, EVs are emerging as powerful diagnostic tools. Their presence in accessible biofluids and their ability to carry disease-specific RNA, protein, lipid, and microbial signatures position them as attractive “liquid biopsy” candidates. EV-based biomarkers correlate with disease severity, inflammatory endotypes, and functional outcomes, and may provide more direct insight into tissue-level processes than conventional clinical parameters. Moreover, microbial EV profiling introduces a novel dimension by linking host immune responses with microbiome composition.

Despite these advances, several challenges remain before EV-based strategies can be fully translated into clinical practice. These include standardization of EV isolation and characterization methods, identification of disease- and cell-specific EV subsets, scalability of EV production, and rigorous evaluation of long-term safety. Furthermore, given the heterogeneity of allergic diseases, future research must focus on defining EV signatures specific to distinct endotypes and developmental stages, as well as on integrating EV-based diagnostics and therapies with existing precision medicine frameworks. In conclusion, EVs dual capacity to act as mediators of pathology and as tools for diagnosis and therapy places them at the forefront of next-generation approaches to allergy research and clinical management. Continued interdisciplinary efforts combining immunology, cell biology, bioengineering, and clinical sciences will be essential to harness the full potential of EVs in preventing, diagnosing, and treating allergic diseases.

## Data Availability

No datasets were generated or analysed during the current study.

## Abbreviations

AD: Atopic dermatitis
ADSC: Adipose-derived mesenchymal stem cell
APC: Antigen-presenting cell
AR: Allergic rhinitis
AREG: Amphiregulin
AS: Atopic asthma
ASC: Human adipose tissue-derived mesenchymal stem cell
AYC-EV: *Aster yomena*- derived EV
BALF: Bronchoalveolar lavage fluid
BEC: Bronchial epithelial cells
BLG: Bovine β-lactoglobulin
BM: Biomarker
BMMC: bone marrow–derived mast cells
CF: Cystic fibrosis
CMA: Cow’s milk allergy
COPD: chronic obstructive pulmonary disease
CR: Chronic rhinitis
CRSwNP: Rhinosinusitis with nasal polyps
CRS: Chronic rhinosinusitis
CSE: Cigarette smoke extract
DC: Dendritic cell
DE: Agricultural dust
DFR: Dupilumab facial redness
DNCB: 1-chloro-2,4-dinitrobenzene
DTH: Delayed-type hypersensitivity
DUSP1: Dual Specificity Protein Phosphatase 1
EA: Eosinophilic asthma
ECM: Extracellular matrix
EcN EV: *E. coli* Nissle 1917- derived EV
EcoR12 EV: *E. coli* strain EcoR12-derived EV
ELISA: Enzyme-Linked Immunosorbent Assay
EPO: Eosinophil peroxidase
EV: Extracellular vesicle
FA: Food allergy
FEV: Forced expiratory volume
FoxP3: Forkhead box P3
GATA3: GATA binding protein 3
HBEC: Human bronchial epithelial cells
HC: Healthy controls
HDM: House dust mite
HDFn: Human neonatal dermal fibroblast
HMGCR: HMG-CoA reductase
hUCMSC: Human umbilical cord-derived mesenchymal stem cells
IEC: Intestinal epithelial cell
IFN-γ: Interferon gamma
IgA/IgE/IgG/IgM: Immunoglobulin A / E / G / M
IL-1, IL-2, ..., IL-33: Interleukin 1, 2, ..., 33
ILC2: Group 2 innate lymphoid cell
iPSC: Induced pluripotent stem cell
JAK: Janus kinase
JNK: c Jun N-terminal kinase
LC: Langerhans cell
LpEV: *Lactobacillus paracasei*- derived EV
LPS: Lipopolysaccharide
LTA: Lipoteichoic acid
M1, M2: Macrophage 1, 2
MAPK: Mitogen-activated protein kinase
MBP1: Major basic protein
MDDC: Monocyte-derived dendritic cells
MHC I, MHC II: Major histocompatibility complex class I, class II
MIEV: *Micrococcus luteus*- derived EV
miRNA: MicroRNA
MISEV: Minimal Information for Studies of Extracellular Vesicles
MS: Mass Spectrometry
MSA: Moderate-to-severe asthma
MSC: Mesenchymal stem cell
mTOR: Mammalian target of rapamycin
NA: Neutrophilic asthma
NEA: Noneosinophilic asthma
NF-κB: Nuclear factor kappa-B
NO: Nitric oxide
NOD: Nucleotide-binding oligomerization domain
NV: EV-mimetic nanovesicle
OVA: Ovalbumin
PBMC: Peripheral blood mononuclear cells
PI3K/AKT: Phosphoinositide 3-kinase/protein kinase B
ROS: Reactive oxygen species
RT qPCR: Reverse transcription quantitative polymerase chain reaction
SC: Stratum corneum
SCIT: Subcutaneous immunotherapy
SE-EV: *Staphylococcus epidermidis*- derived EV
sEVs: Small extracellular vesicle
SHS: Second-hand smoke
SSRA: Steroid-resistant asthma
STAT3, STAT6: Signal transducer and activator of transcription 3, 6
TCR: T-cell receptor
TGF-β: Transforming growth factor beta
Th1/Th2/Th17: T helper cell types 1 / 2 / 17
TLR: Toll-like receptor
TNF-α / TNF-β: Tumor necrosis factor alpha / beta
Treg: Regulatory T cell
TSLP: Thymic stromal lymphopoietin
